# Early animal evolution and highly oxygenated seafloor niches hosted by microbial mats

**DOI:** 10.1038/s41598-019-49993-2

**Published:** 2019-09-20

**Authors:** Weiming Ding, Lin Dong, Yuanlin Sun, Haoran Ma, Yihe Xu, Runyu Yang, Yongbo Peng, Chuanming Zhou, Bing Shen

**Affiliations:** 1 Key Laboratory of Orogenic Belts and Crustal Evolution, MOE, Beijing 100871 China; 2 School of Earth and Space Science, Peking University, No. 5 Yiheyuan Road, Haidian District, Beijing 100871 China; 3 Department of Geology and Geophysics, Louisiana State University, Baton Rouge, Louisiana 70803 USA; 4 Key Laboratory of Economic Stratigraphy and Palaeogeography, Nanjing Institute of Geology and Palaeontology, Chinese Academy of Sciences, Nanjing, 210008 China

**Keywords:** Biogeochemistry, Biogeochemistry

## Abstract

The earliest unambiguous evidence for animals is represented by various trace fossils in the latest Ediacaran Period (550–541 Ma), suggesting that the earliest animals lived on or even penetrated into the seafloor. Yet, the O_2_ fugacity at the sediment-water interface (SWI) for the earliest animal proliferation is poorly defined. The preferential colonization of seafloor as a first step in animal evolution is also unusual. In order to understand the environmental background, we employed a new proxy, carbonate associated ferrous iron (Fe_carb_), to quantify the seafloor oxygenation. Fe_carb_ of the latest Ediacaran Shibantan limestone in South China, which yields abundant animal traces, ranges from 2.27 to 85.43 ppm, corresponding to the seafloor O_2_ fugacity of 162 μmol/L to 297 μmol/L. These values are significantly higher than the oxygen saturation in seawater at the contemporary atmospheric *p*O_2_ levels. The highly oxygenated seafloor might be attributed to O_2_ production of the microbial mats. Despite the moderate atmospheric *p*O_2_ level, microbial mats possibly provided highly oxygenated niches for the evolution of benthic metazoans. Our model suggests that the O_2_ barrier could be locally overcome in the mat ground, questioning the long-held belief that atmospheric oxygenation was the key control of animal evolution.

## Introduction

The last 10 million years of the Ediacaran Period (550–541 Ma), at the eve of Cambrian Explosion, experienced dramatic and enigmatic changes in the biosphere^1,2^. The classic Ediacaran biota showed a sharp decline in diversity^3^, and the earliest unambiguous bilaterian animals—represented by various types of trace fossils^4,5^—began to occupy ecological niches on the seafloor. For example, the first U-shaped trace fossil, *Arenicolites* from Western Mongolia, indicates that early animals had the ability to burrow vertically into sediments^6^, and the oldest trackways from South China imply the presence of bilaterian animals with paired appendages^7^.

Recent Uranium (U) isotope studies indicated there was extensive oceanic anoxia (>21% of the seafloor) during terminal Ediacaran, which was associated with the decline of Ediacaran biota^8^. There are also previous studies suggesting that, although atmospheric *p*O_2_ level remained modest, varying between 10–40% Present Atmospheric Level (PAL)^9,10^, the deep ocean remained predominantly anoxic^11^, and the ocean oxidation was episodic throughout the Ediacaran and Cambrian^12,13^. The full oxygenation of the ocean-atmosphere system did not occur until the latter Pleozoic^9^. Although some geochemical evidences have been interpreted as increased oxygenation during the terminal Ediacaran^14–16^, redox conditions adjacent to the sediment-water interface (SWI) is still poorly constrained.

The O_2_ fugacity of the modern seafloor is highly heterogeneous (0 ~ >250 μmol/L)^17^, suggesting that atmospheric oxygenation and seawater oxidation does not necessarily mean high seafloor O_2_ fugacity. Existing geochemical proxies, such as Fe-S-C systematics^12,18,19^, molybdenum isotopes^20^, Ce anomalies^21^, nitrogen isotopes^22^ and abundance of redox-sensitive trace elements^23^, reflect the degree of seawater oxidation, but make no direct inference about the seafloor redox. The earliest mobile animals, represented by the occurrence of Ediacaran and early Cambrian trace fossils, predominantly have benthic rather than pelagic lifestyle^24,25^. Yet, it remains unclear what exactly were the oxygen conditions under which the animals, especially benthos that live on the SWI or even penetrate into the sediment, survived and radiated in the terminal Ediacaran ocean.

**Figure 1:**
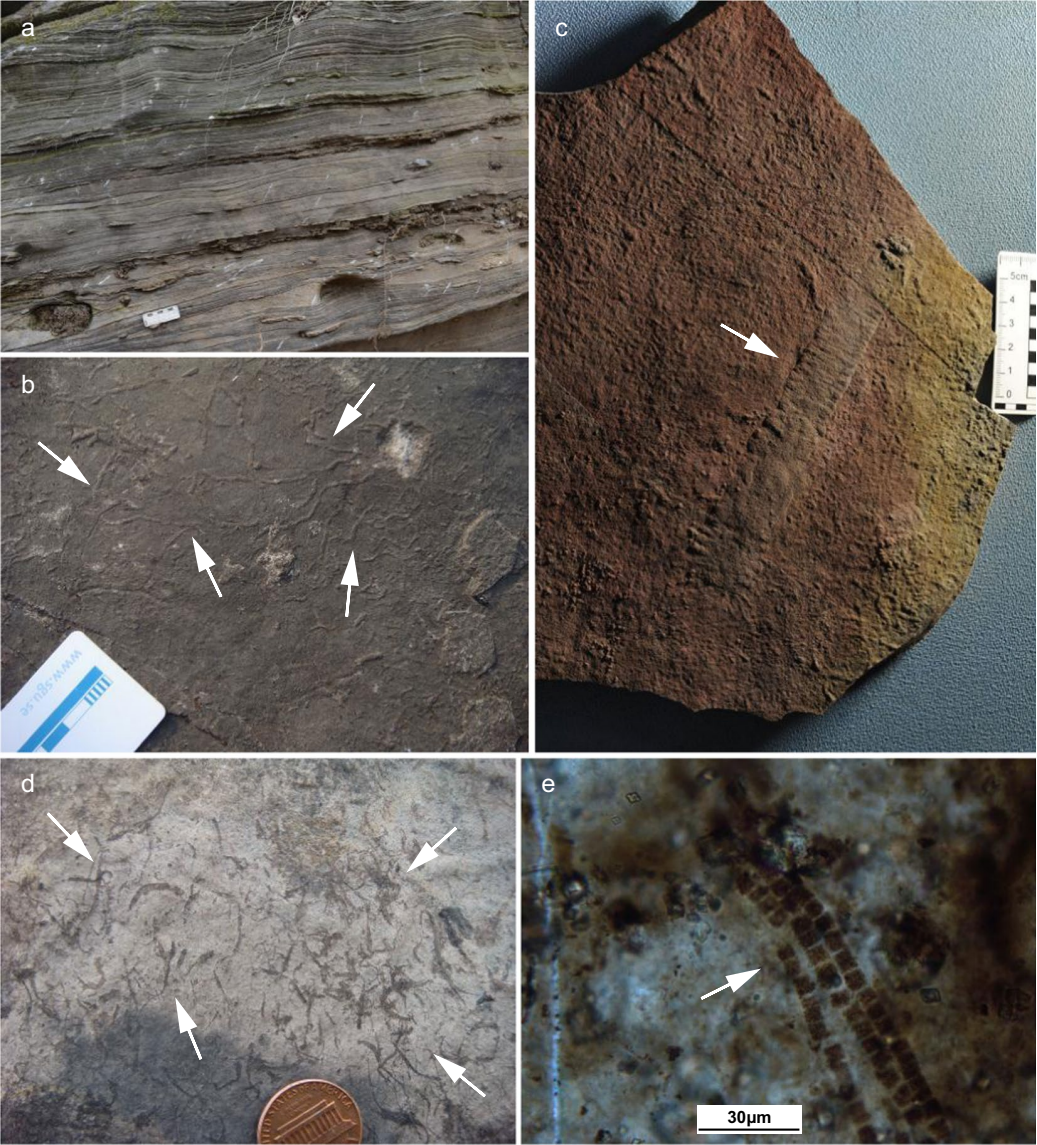
Lithology and fossils of the Shibantan Member, Dengying Formation. (**a**) Dark, laminated organic-rich limestone; (**b**) Abundant trace fossils (white arrows); (**c**) *Wutubus annularis*. White arrow points to apex; (**d**) *Vendotaenia* (white arrows); (**e**) Typical benthic cyanobacteria *Oscillatoria* from the chert nodules in the Denying limestone (white arrow).

In order to understand the environmental conditions experienced by the earliest benthic animals, we propose a method of retrieving the seafloor O_2_ fugacity from sections where the earliest animals evolved. The Ediacaran Dengying Formation (551–541 Ma), Yangtze Block, South China, contains abundant trace fossils together with both canonical and atypical Ediacaran fossils^4,26,27^. These fossils are particularly well known from in the bituminous limestone of the Shibantan Member (Fig. 1). In addition, the earliest biomineralized organism, *Cloudina*, has been discovered in the Baimatuo Member of the uppermost Dengying Formation^28–31^. As one of the few terminal Ediacaran fossiliferous carbonate successions in the world, the Dengying limestone may provide a unique window to view the environmental and ecological background of the latest Ediacaran evolution. In this study, we developed a new proxy, carbonate associated ferrous iron (Fe_carb_), to constrain the seafloor O_2_ fugacity during the emergence of the earliest benthic animals in the latest Ediacaran Dengying Formation in South China.

## Fe_carb_ As A Proxy of Seafloor O_2_ Fugacity

Ferrous Fe [Fe(II)] is thermodynamically unstable in oxic conditions, and would be oxidized to ferric Fe [Fe(III)], resulting in the precipitation of iron oxides or iron oxyhydroxides at neutral to alkaline pH conditions. The redox equilibrium between Fe^2+^ and O_2_ could be expressed as:

1 $$4F{e}^{2+}+{O}_{2}+10{H}_{2}O\to 4Fe{(OH)}_{3}+8{H}^{+}$$

Thus, modern oxic seawater have extremely low dissolved Fe content (0~1.5 nmol·L^−1^)^32^. Ferric Fe can be reduced by iron reducing microbes (IRM) in suboxic-anoxic sediments by process collectively known as the dissimilatory iron reduction (DIR)^33^. The chemical reaction of DIR can be expressed as:

2 $$2F{e}_{2}{O}_{3}+C{H}_{2}O+3{H}_{2}O\to 4F{e}^{2+}+HC{O}_{3}^{-}+7O{H}^{-}$$

DIR generates Fe^2+^, increasing Fe^2+^ concentration in sediment porewater (0~500 μmol·L^−1^ in modern ocean)^34,35^. As such, high porewater Fe^2+^ content and low seawater Fe^2+^ content generate a Fe^2+^ concentration gradient, which results in the upward diffusion of dissolved Fe^2+^ and a benthic Fe^2+^ flux. The concentration gradient of Fe^2+^ (∇_*Fe*_) can be expressed by the following simplified equation:

3 $${\nabla }_{Fe}=\frac{({[Fe]}_{pw}-{[Fe]}_{sw})}{{l}_{Fe}}$$

where l_Fe_ is the depth of the upper boundary of DIR zone below SWI. [Fe]_pw_ and [Fe]_sw_ are Fe^2+^ concentration in porewater (within the DIR zone) and seawater, respectively. [Fe]_pw_ is determined by the availability of ferric Fe oxides/oxyhydroxides and organic matter (Eq. 2). Considering the large difference in the order of magnitudes between [Fe]_pw_ and [Fe]_sw_, the concentration gradient is mainly controlled by [Fe]_pw_ as follows:

4 $${\nabla }_{Fe}=\frac{{[Fe]}_{pw}}{{l}_{Fe}}$$

The benthic flux of Fe^2+^ (Flux_Fe_) can be expressed as:

5 $$Flu{x}_{Fe}={\nabla }_{Fe}\times {D}_{Fe}$$

where D_Fe_ is the diffusivity coefficient of Fe^2+^ in porewater. At equilibrium, Eq. 1 can be written as:

6 $$K=\frac{{[{H}^{+}]}^{8}}{{[F{e}^{2+}]}^{4}\times [{O}_{2}]}$$

where K is the equilibrium constant. The Fe^2+^ concentration shows an inversely exponential relationship with the O_2_ content. Rearranging Eqs 4–6, we arrive at:

7 $$Flu{x}_{Fe}={D}_{Fe}\times \frac{{[{H}^{+}]}^{2}}{{l}_{Fe}\times {(K\times [{O}_{2}])}^{1/4}}$$

Thus, in theory, when pH and temperature remain unchanged, there is an inversely exponential relationship between benthic iron flux (Flux_Fe_) and bottom water O_2_ (O_2-BW_) adjacent to the SWI, which can be expressed as an empirical equation:

8 $$Flu{x}_{Fe}=a\times {({O}_{2-BW})}^{b}$$

In the modern ocean, there exists a negative correlation between O_2-BW_ and Flux_Fe_^36–38^ (Supplementary Fig. 6). Instead of using *in situ* fluxes, we collected the benthic flux data measured by non-invasive benthic chambers^39^. Strong benthic bioturbation related with water depth in the modern ocean can elevate the iron flux (Supplementary Fig. 6). In order to recede the influence of benthos, we choose the data collected from locations with water depth greater than 500 m. Thus, the best fitted power function can be expressed as follows (see supplementary text; Supplementary Fig. 7; the units of Flux_Fe_ and O_2-BW_ are mol · m^−2^ · Myr^−1^ and mol · L^−1^):

9 $$Flu{x}_{Fe}={10}^{-4.98\pm 0.72}\times {({O}_{2-BW})}^{-1.71\pm 0.16}$$

We suggest that Flux_Fe_ could be recorded by carbonate precipitating on the seafloor. Because Fe^2+^ and Ca^2+^ have similar ionic radii and charge, Fe^2+^ has the tendency to replace Ca^2+^ in carbonate minerals^40^. Fe_carb_ is determined by Fe^2+^ concentration in solution that is related to the redox condition (or oxygen fugacity) and the partitioning coefficient that is temperature, pH, Eh and mineralogy dependent^41^. Because [Fe]_pw_ is at least 3 orders of magnitude larger than [Fe]_sw_, benthic flux (with Fe^2+^ flux of 0.02~568 μmol · m^−2^ · d^−1^ in the modern ocean) would be the major Fe source for benthic carbonates. Equation for Fe_carb_ of seafloor carbonate precipitation can be expressed as (see supplementary text):

10 $$F{e}_{carb}=\frac{{K}_{Fe}\times {M}_{Fe}\times {10}^{-4.98}\times {({O}_{2-BW})}^{-1.71}}{s\times \rho }$$

where K_Fe_ is the partitioning coefficient of the benthic Fe^2+^ flux into the carbonate lattice. M_Fe_ is the molecular weight of Fe (56 g/mol), s is the sedimentation rate, and ρ is the density of carbonates (2.71 g/cm^3^).

Notably, although Fe_carb_ content is determined by the seafloor O_2_ fugacity, which is controlled—although not uniquely—by the atmospheric *p*O_2_ level, the quantitative reconstruction of atmospheric *p*O_2_ level by using Fe_carb_ is not directly applicable. On one hand, Fe speciation in seawater is complex. In addition to free Fe^2+^, the dissolved ferrous Fe species also include various Fe-organic complexes, which accounts for the majority of the dissolved Fe in the modern ocean^32^. On the other hand, atmospheric *p*O_2_ level is not the only control of bottom seawater redox. Instead, both organic matter influx and ocean circulation also play important roles^42^. If the water column above the SWI enriches organic matter or ocean circulation is stagnant, there can be decoupling between atmospheric *p*O_2_ level and bottom water O_2_ content. Therefore, Fe_carb_ can only constrain the redox conditions on the seafloor, and not in the atmosphere.

**Figure 2:**
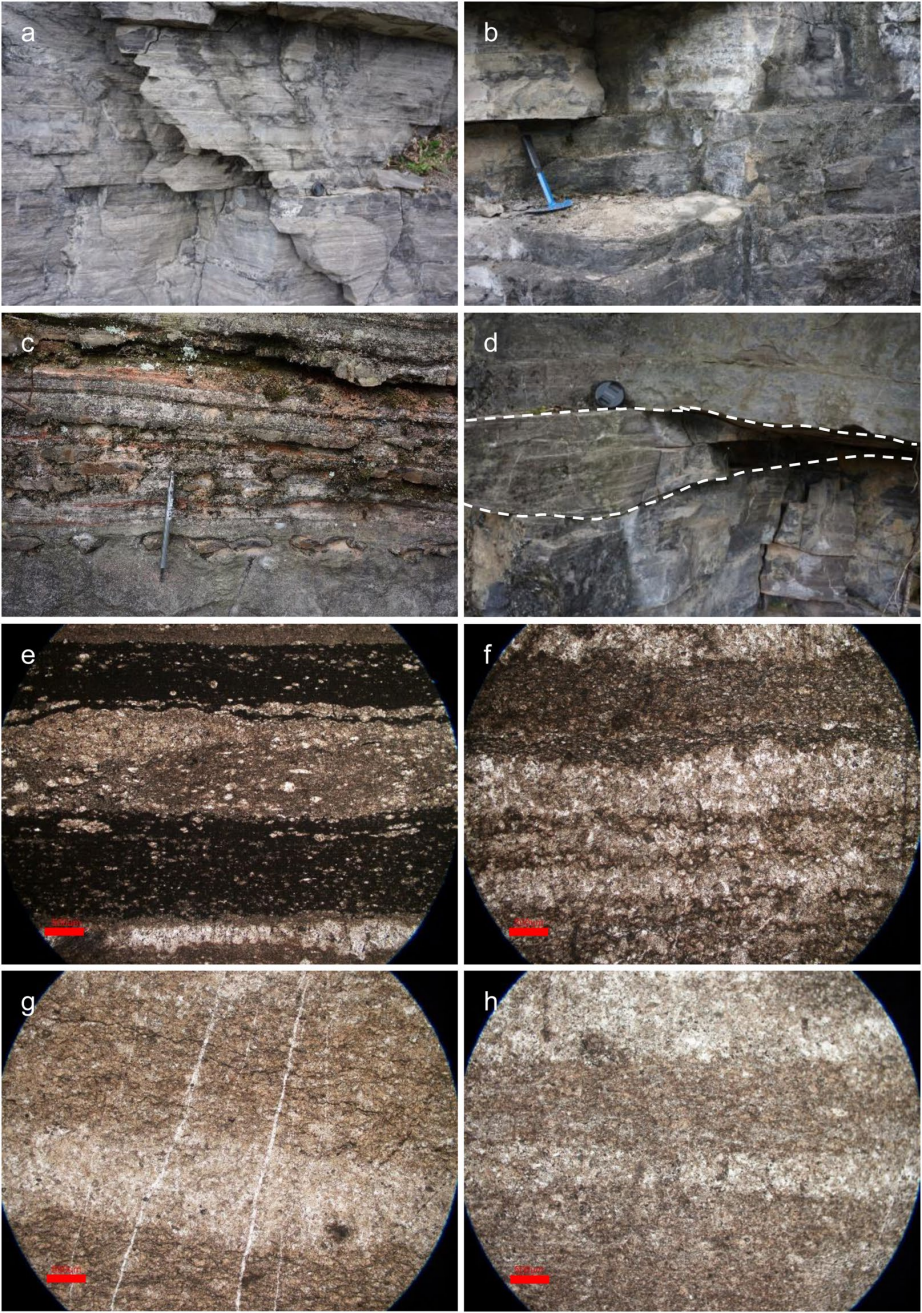
Field photographs and transmitted light photomicrographs. (**a**) Outcrop photograph showing the well-laminated limestone of the Shibantan Member, Dengying Formation. (**b**) Outcrop photograph showing dark-colored organic rich limestone. (**c**) Outcrop photograph showing the chert nodules in the limestone of the Shibantan Member, Dengying Formation. (**d**) Outcrop photograph showing hummocky cross beddings (dashed lines for orientation). (**e**,**f**) Photomicrograph showing organic rich micro-laminae of micrite and calcspar. Several calcspar-grains can be seen in the micritic layer. (**g**,**h**) photomicrograph showing alternating calcspar and micritic layers. The boundary between the laminae is fuzzy. Scale bars are 500 μm for pictures e–h.

Furthermore, using Fe_carb_ to reconstruct seafloor O_2_ fugacity can only be applied to carbonate that precipitated on the seafloor. Before the evolution of skeletonizing organisms, i.e. Ca-carbonate biomineralization in the latest Ediacaran^43,44^, marine carbonate precipitation derive from biotically induced carbonate precipitation and direct abiotic precipitation from seawater or porewater^45^. The inorganic precipitation, identified by crystal fan and herringbone structures in carbonate, was common in Archean and decreased in abundance after the late Paleoproterozoic^46^. By contrast, biotically induced precipitation is driven by an elevation of carbonate saturation resulting from releasing of microbial metabolite into carbonate producing micro-environments^47,48^. The Shibantan limestone contains abundant organic-rich filaments, and is composed of crinkled microlaminae that have been interpreted as microbial mats^7,27,49^ (Fig. 2). It is reasonable to argue that the Dengying carbonate precipitation was triggered by benthic microbes on the seafloor, warranting the application of Fe_carb_ to reconstruct seafloor O_2_ fugacity. Furthermore, before the evolution of pelagic planktonic carbonate secreting organisms in Mesozoic, nearly all marine carbonate in the Paleozoic ocean was generated by benthic calcifiers, such as brachiopods, corals, and echinoderms^50^. Although carbonates precipitation from the water column cannot be completely ruled out, physical and biological abrasions of biogenic carbonate should be the major source of carbonate mud (i.e. micrite) in the Paleozoic ocean^51^. Therefore, Fe_carb_ of micrite from the Paleozoic carbonate can be used to reconstruct the seafloor O_2_ fugacity as well. In this study, we use Fe_carb_ of the late Paleozoic (late Devonian and early Carboniferous) limestone (see supplementary text; Supplementary Fig. 3) as references. It is reasonable to speculate that the concentration of dissolved oxygen in the late Paleozoic shallow water was in equilibrium with the atmosphere, whose *p*O_2_ levels were at least comparable to or even higher than that of the modern atmosphere^9^.

It should be noted that there are limitations and assumptions when applying Fe_carb_ to reconstruct the seafloor O_2_ fugacity. First, considering the short residence time of dissolved iron in seawater (on the order of 100~200 yr)^52^, Fe_carb_ only reflects the local seafloor redox rather than the global state which can be estimated by uranium isotopes^53^. Second, Eq. 10 is based on the assumption of unlimited benthic Fe^2+^ flux. However, benthic Fe^2+^ flux would be finite when there are deficient supplies of reactive Fe or organic matter. In this case, low Fe_carb_ could also be generated at low seafloor O_2_ fugacity with insufficient supplies of reactive Fe or organic matter. Thus, the interpretation of Fe_carb_ data should also consider siliciclastic and TOC contents in carbonate so as to guarantee sufficient Fe flux from sediment porewater. Third, we suggest that Fe_carb_ can only be applied to limestone rather than dolostone. Generally higher Fe_carb_ of dolostone may not only be the consequence of Fe-enriched dolomitization fluid, but also result from higher miscibility between Mg and Fe in carbonate lattice than between Ca and Fe^40^. Possibly multiple stages and fluid origins of dolomitization also interfere Fe_carb_ as a seafloor redox indicator. Thus, we recommend samples with low Mg/Ca (<0.05) should be selected. Finally, authigenic carbonate precipitation within DIR zone that has high Fe^2+^ content could also contribute to higher Fe_carb_. Therefore, Fe_carb_ represents the minimum estimation of the seafloor O_2-BW_.

## Geological Setting and Sample Description

The Dengying Formation in the Yangtze Gorges area can be divided into, in ascending order, the Hamajing, Shibantan and Baimatuo members^30^ (Supplementary Fig. 2). The Shibantan Member, sandwiched between the peritidal dolostone of the Hamajing and Baimatuo members, is composed of dark, laminated, organic-rich limestone. The Shibantan limestone contains a variety of fossils, including trace fossils, Ediacara fossils (e.g. *Wutubus annularis*), algal fossils (e.g. *Vendotaenia*), as well as benthic cyanobacteria (e.g. *Oscillatoria*) (Fig. 1). The absence of subaerial exposure structures as well as the occasional occurrences of hummocky cross bedding suggests the deposition in a deep subtidal environment, probably below the fair-weather wave base but above the storm-wave base (Fig. 2; see Supplementary Text).

Samples were collected from the Sixi and Huangniuyan sections in the Yangtze Gorges area, South China (Fig. 2a–d; Supplementary Fig. 1). The fine laminae are confined by organic-rich microbial filaments and are composed of alternating micritic and calcspar layers (Fig. 2e–h). The micritic layer normally has higher organic and siliciclastic contents, while the calcspar layer is composed of subhedral-anhedral calcite crystals of up to 100 μm in size. The calcspar crystals usually have fuzzy boundaries and contain abundant remaining micrites, suggesting the calcspar might derive from recrystallization of micrite in the early stage of diagenesis.

## Results

**Figure 3:**
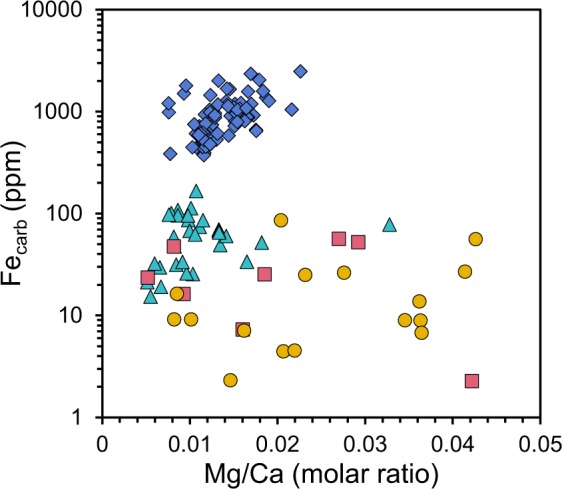
Fe_carb_ concentration of the Dengying Formation and carbonates in late Paleozoic including samples with Mg/Ca < 0.05. Crossplot showing Mg/Ca molar ratio(x-axis) vs. Fe_carb_ content(y-axis). Low Fe_carb_ content of micrite (red squares) and calcispar (yellow dots) layers of the Dengying Formation shows no significant difference, indicating that carbonates precipitated in Fe-depleted condition. Fe_carb_ content of shallow water carbonates in late Paleozoic (cyan triangles) is comparable to that of the Dengying Formation. Deep water carbonates in late Paleozoic (blue rhombuses) are characterized by high Fe_carb_.

The micrite and calcspar layers of the Shibantan limestone have comparable Fe_carb_, ranging from 2.27 ppm to 157.34 ppm (mean = 47.49 ppm, n = 22) and from 2.31 ppm to 260.15 ppm (mean = 57.12 ppm, n = 32), respectively (Supplementary Fig. 4; Supplementary Table 1). The limestone samples with Mg/Ca molar ratio < 0.05 have Fe_carb_ varying between 2.27 ppm and 85.43 ppm (mean = 22.48 ppm, n = 24) (Fig. 3). As a comparison, Fe_carb_ of the late Paleozoic carbonates shows a wide range of variation (Fig. 3; Supplementary Table 2). In general, samples from shallow water carbonate platform environment, including the Panlong, Madao and Dazhai sections, have lower Fe_carb_, ranging from 15.28 ppm to 166.26 ppm (mean = 63.44 ppm, n = 31), whereas the equivalent deep water samples from the Duli, Xiada and Daposhang sections have significantly higher Fe_carb_, varying between 373.01 ppm and 2471.23 ppm (mean = 924.42 ppm, n = 83) (Fig. 3; Supplementary Table 2).

## Discussion

The Shibantan limestone (Mg/Ca molar ratio < 0.05) samples have extremely low Fe_carb_ values both in micritic and calcspar layers, with little difference between these two types of layers, supporting the petrological observation that the calcspar mainly derives from recrystallization of micrite (Fig. 3). In addition, the Mn content in the Shibantan limestone is extremely low or even undetectable, suggesting little alteration by meteoric fluids^54^ (Supplementary Table 1). Other diagenetic processes, which dominantly occur in anoxic conditions and cause more Fe^2+^ incorporation into the carbonate lattice, would most likely elevate Fe_carb_. Therefore, low Fe_carb_ of the Dengying limestone may not result from diagenetic processes.

Low Fe_carb_ of the Dengying limestone cannot be attributed to oceanic euxinia as well (i.e. H_2_S enriched but Fe^2+^ depleted), because abundant trace fossils and macroscopic Ediacara fossils strongly argue for a non-sulfidic environment^4,26,49^. Neither could the low Fe_carb_ be attributed to low [Fe]_pw_ resulting from insufficient supply of organic matter and reactive Fe. Firstly, the Dengying limestone has high siliciclastic contents (average = 14.34%, n = 47; Supplementary Table 4), suggesting sufficient reactive Fe in sediments (Supplementary Fig. 9). Secondly, high organic carbon content (average = 2.47%, n = 47, Supplementary Table 4) in bituminous limestone warrants DIR in sediment porewater. In addition, low Fe_carb_ of the Shibantan limestone (mean = 22.48 ppm, n = 24) is close to, or even lower than that of the late Paleozoic shallow water carbonates (mean = 63.44 ppm, n = 31; Fig. 3). Considering the sedimentation rates of the Shibantan limestone (at least 24 m/Myr) and shallow marine carbonates in the Late Paleozoic sections (12.5 m/Myr for the Madao section, 6.4 m/Myr for Panlong section and 28.6 m/Myr for Dazhai section), the Ediacaran seafloor O_2_ fugacity should be comparable to that of the well ventilated seafloor in Late Paleozoic (Eq. 10). Thus, low Fe_carb_ of the Shibantan limestone can only be explained by high seafloor O_2_ fugacity.

**Figure 4:**
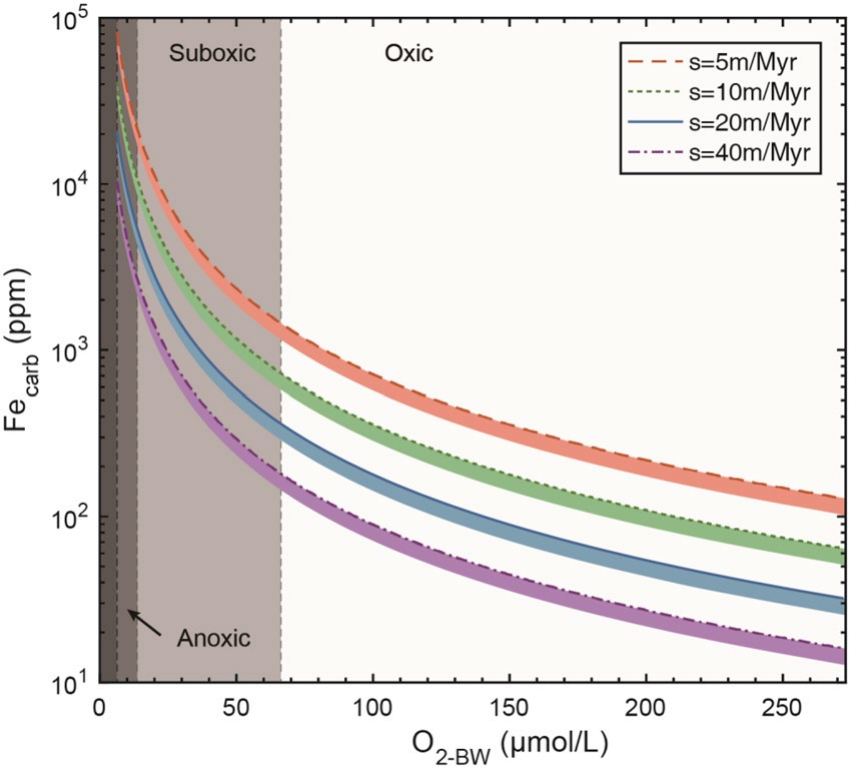
Modelling results according to the Eq. 10. Fe_carb_ trapped in the carbonate lattice (y-axis) varies as a function of the bottom water oxygen level (O_2-BW_, x-axis). Contour lines indicate different sedimentation rate of carbonates. The lower limit of O_2-BW_ was set at 6.25 μmol/L, the boundary of euxinia in the water column^21^. The upper limit of O_2-BW_ was set at 273 μmol/L, the saturated O_2_ level in the water column. Vertical dashed lines define the boundary of dissolved O_2_ level for oxic (>68 μmol/L), suboxic (13.6 μmol/L~68 μmol/L) and anoxic (6.25 μmol/L~13.6 μmol/L) conditions^49,50^. The color areas suggest the possible relationship between Fe_carb_ and O_2-BW_ if we assume that 20% of carbonate formed in the water column. See texts for more information.

To quantify the seafloor O_2_ fugacity by Eq. 10, K_Fe_ should be determined in advance. However, K_Fe_ that specifically represents partition coefficient of benthic Fe^2+^ incorporation into calcite has not been directly determined for modern limestone, although the factors affecting Fe^2+^ incorporation into calcite in aqueous solution at equilibrium state have been investigated^55,56^. To constrain this unknown, we use Fe_carb_ data of late Devonian-early Carboniferous shallow marine carbonate samples (Madao, Panlong and Dazhai sections), to calculate the K_Fe_. The calculated K_Fe_ value is then validated by the equivalent deep water carbonate samples (Duli, Xiada and Daposhang sections), which were collected from the beddings that contains abundant benthic animal fossils and thus were also inferred to represent oxic conditions (Fig. 3; see supplementary text; Supplementary Table 2). The result shows that the average K_Fe_ is 2.32 (1.86 for Madao section, 1.89 for Panlong section and 3.22 for Dazhai section). These calculated K_Fe_ values are within the range of values previously determined by experimental studies (1.5–2.3 at 10 °C and 2.8–7.7 at 50 °C)^55^. To reconstruct O_2-BW_ during the deposition of carbonates by Eq. 10, we use K_Fe_ = 2.32 and s = 5, 10, 20, 40 m/Myr. O_2-BW_ of 6.25 μmol/L is set as the upper bound of anoxic and euxinic conditions (i.e. microbial sulfate reduction occurs below this threshold)^17^ and 68 μmol/L as the cutoff for the suboxic and oxic conditions^57,58^. The modeling result displays a negatively exponential relationship between Fe_carb_ and O_2-BW_ (Fig. 4). Indeed, we cannot completely exclude carbonate precipitation from the water column, which will ultimately decrease Fe_carb_ due to low Fe^2+^ content in the seawater. Assuming that 80% of carbonate originally precipitates on the seafloor, and the other 20% precipitates from the water column, the colored area in Fig. 4 indicate the range of Fe_carb_ and O_2-BW_ changes under different sedimentation rates.

Thus, the calculated O_2-BW_ (average Fe_carb_ of 22.48 ppm for the Dengying limestone), i.e., the seafloor O_2_ fugacity during terminal Ediacaran, ranges from 162 μmol/L to 297 μmol/L, when the sedimentation rate varies between 24 m/Myr and 67 m/Myr (see supplementary text; Supplementary Table 3). The upper bound (297 μmol/L) exceeds the saturated dissolved O_2_ of 273 μmol/L in seawater at 1 PAL *p*O_2_, while the lower bound (162 μmol/L) is equivalent to the dissolved O_2_ content at 60% PAL *p*O_2_. If we assume that measured Fe_carb_ of the Dengying Formation is 20% lower than original benthic carbonates because of seawater carbonate mixing, the minimum seafloor O_2_ fugacity is still 143 μmol/L. Thus, even if the Ediacaran atmospheric *p*O_2_ level reaches the maximum estimate of 40% PAL^9^, the seafloor O_2_ fugacity during the deposition of the Shibantan limestone was likely oversaturated with respect to the atmospheric *p*O_2_ level.

**Figure 5:**
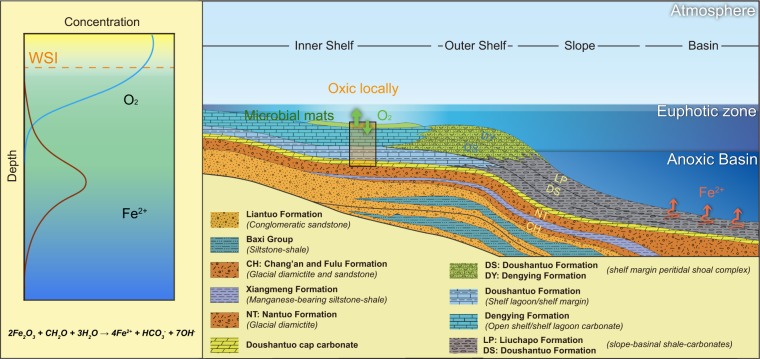
Schematic diagram illustrating the depositional environment of Dengying Formation (modified according to previous literatures^64,65^). The O_2_ level in the atmosphere remained 10–40% PAL. The interface between atmosphere and water column could be slightly oxidized by the O_2_ dissolution, whereas the majority part of the ocean still remained anoxic. Microbial mats, colonizing the seafloor where the Shibantan Member deposited, produced O_2_ as benthic photosynthesis organism, which supersaturated the adjacent water column with O_2_. In addition, the downward diffusion of O_2_ would lower the redox boundary of iron reduction, reducing the benthic flux of Fe^2+^.

**Figure 6:**
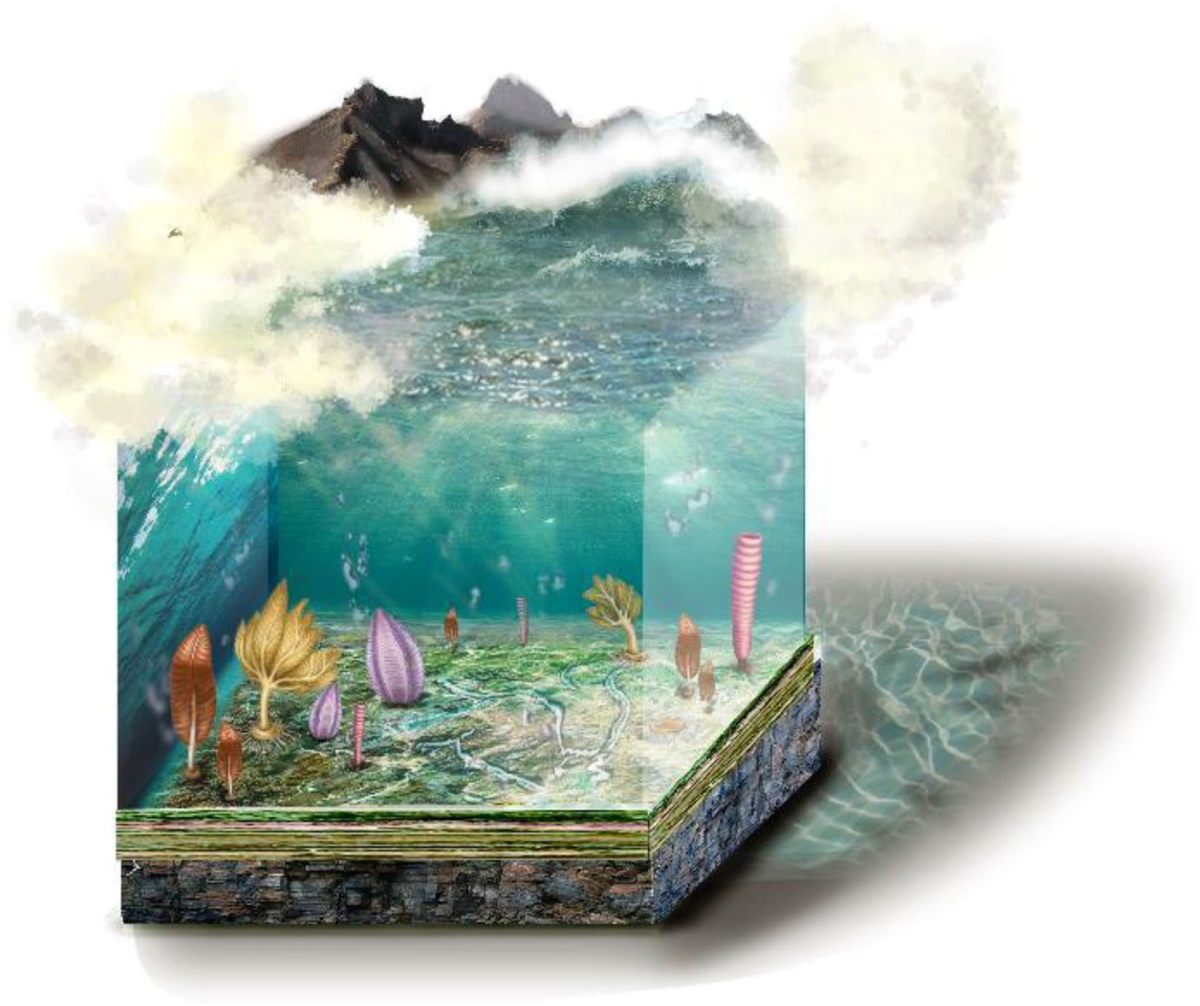
Schematic diagram illustrating the biological oasis provided by microbial mats. O_2_ bubbles produced by microbial mats diffused all around, making the ajancent water column partially oxidized. Oxygenation of uppermost sediments by downward diffusion of O_2_ provided the niches for high ecological complexity of benthic communities. The Ediacara biota in the diagram all discovered in the Shibantan Member, Dengying Formation^4,26^.

High seafloor O_2_ fugacity during the deposition of the Shibantan limestone seems contradictory with the modest atmospheric *p*O_2_ levels and extensive seafloor anoxia in Ediacaran and Cambrian^8,59^. Local seafloor oxygenation must require a continuous O_2_ supply to maintain oxic status under such predominately anoxic condition. Inspired by a modern analog, Los Roques lagoon in Venezuela^60^ has low O_2_ concentration in water column and is colonized by O_2_-generating cyanobacteria mat in floor, but the O_2_ concentration in the mat ground could be four times higher than that in the water column. Here, we suggest that the seafloor oxygenation might result from the development of microbial mats on the seafloor during the precipitation of Shibantan limestone (Figs 1a,e, 5 and 6). Microbial mats generate O_2_ that is ready to be emitted into the water column, resulting in partial oxygenation of the adjacent bottom water. In addition, the downward diffusion of O_2_ produced by microbial mats would lower the redox boundary of DIR zone, reducing the intensity of benthic flux of Fe^2+^ (Fig. 6). This model is consistent with the widespread microbial structures, e.g. warty textures^26^, microbial laminae^4^, and benthic cyanobacteria (Fig. 1e) in the Dengying limestone.

It has been proposed that microbial mats might have played a key role in the preservation of Ediacara fossils (the death mask hypothesis)^61^, and the disappearance of Ediacara fossils at the Ediacaran-Cambrian boundary might be related to the disappearance of microbial mats after the evolution of metazoans^2^. Here, our new model proposes an alternative, but not mutually exclusive, interpretation that microbial mats might also provide a more locally oxygenated environment in the context of generally low atmospheric *p*O_2_ level and widespread seafloor anoxia. Thus, microbial mats on shallow marine seafloor may generate an oxygenated oasis that might have stimulated the diversification of metazoans, even when the atmospheric *p*O_2_ level was only 10–40% PAL, barely meeting the threshold for animal evolution^16^. Therefore, it is plausible that the earliest animals would refrain from floating in the ocean that is primarily anoxic and is characterized by dramatic redox oscillation, and prefer utilizing O_2_ and food on and within the microbial mat. Such a hypothesis is also supported by the widespread late Ediacaran trace fossils associated with microbial sedimentary structures^4,5^, some of which may indicate activities under microbial mats^49^. Our hypothesis could also support that the majority of the earliest animals were evidenced by trace fossils which record benthic instead of pelagic ethology. The lack of pelagic body fossil records may reflect the ecological constraint of the terminal Ediacaran communities driven by the ocean redox, not just a result of taphonomy or poor preservation.

Finally, the rise of atmospheric *p*O_2_ level, which was thought to provide the upper constraint on the redox of the ocean, has been regarded as the *priori* for the animal evolution. However, our study suggests that local seafloor O_2_ fugacity might significantly exceed the saturated O_2_ content at a given atmospheric *p*O_2_ level, and the local seafloor oxygenation might be attributed to the development of microbial mats. If this is the case, seafloor oxidation and atmospheric oxygenation might be decoupled. It is highly probable that seafloor might have long been locally oxidized when atmospheric *p*O_2_ level was still low, because oxygenic microbial mats, e.g. stromatolites, are believed to cover the shallow marine seafloor since Archean time^62^. Therefore, we suggest that atmospheric or oceanic oxygenation may not be the crucial control on the emergence of animals; instead, life may have played the central role in the evolution of habitable planet.

## Methods

### Elemental compositions measurement

Mirrored thin and thick sections were prepared for micro-mill sampling. Sample powders were micro-drilled from the thick section under the guide of thin section observation under optical microscopy. Based on the character of laminated limestone, two types of carbonate fabrics, micrite and calcispar, were sampled from the same specimen. About 50 mg of limestone powder was collected approximately in each sample and placed into a 15 ml centrifuge tube.

The sample preparation followed the sequential extraction procedure for carbonated associated Fe designed by Poulton and Canfield (2005)^63^. A buffer solution mixed by acetic acid (HAc) and ammonium acetate (NH_4_Ac) was prepared, and the pH of 4.5 was adjusted accurately before use. For each sample, about 50 mg of sample powder was weighed and was dissolved in 10 ml buffer solution in a centrifuge tube. In order to ensure the solution has full contact with the sample, tubes were placed in a shaking table at 50 °C for 48 hours. After centrifugation, 0.5 ml supernatant was taken out and was mixed with 4.5 ml 2% nitric acid (HNO_3_) in a new centrifuge tube. Finally, elemental compositions were measured with a Spectra Blue Sop Inductively Coupled Plasma Optical Emission Spectrometry (ICP-OES) at Peking University. All analyses were calibrated by a series of gravimetric standards with different concentrations (ranging from 0.1 ppm to 10 ppm) that were run before sample measurements.

### TOC measurement

The limestone of the Dengying Formation was smashed into sample powder and about 100 mg of powder for each sample was weighed and was placed into a 50 ml centrifuge tube. To fully remove the inorganic carbon, 20 ml hydrochloric acid (HCl, 3N) was added to each centrifuge tube, which was then placed in an ultrasonic bath for 1 hour. The reaction was allowed for 12 hours. Then Milli-Q water (18.2 MΩ) was used to rinse the powders until pH reaches 4–5. After that, samples were dried overnight and loaded into capsules for TOC analysis at the Stable Isotope Research Facility (SIRF) at Louisiana State University, USA. Elemental analyzer (Micro Vario Cube, Isoprime Ltd., Cheadle, UK) flash-combust the samples in Tin capsule in a 950 °C furnace. Isoprime 100 (Isoprime 100, Cheadle, UK) gas source mass spectrometer can analyze the resulting CO_2_ by continuous flow. The analyzed precision for TOC data is within 0.3%.

## Supplementary information

Supplementary Information

## Data Availability

All data is available in the main text or the supplementary materials.
