# Supplementary material for: Early animal evolution and highly oxygenated seafloor niches hosted by microbial mats
Source: Sci Rep. 2019 Sep 20;9:13628. doi: 10.1038/s41598-019-49993-2 (PMC6754419; doi:10.1038/s41598-019-49993-2)
Supplement: Supplementary file 1 — Supplementary Information [file 41598_2019_49993_MOESM1_ESM.docx]

**SUPPLEMENTARY INFORMATION TO**

**Early animal evolution and highly oxygenated seafloor niches hosted by microbial mats**

Weiming Ding, Lin Dong, Yuanlin Sun, Haoran Ma, Yihe Xu, Runyu Yang,

Yongbo Peng, Chuanming Zhou, Bing Shen

**This PDF file includes:**

Supplementary text

Supplementary Figure 1-9

Supplementary Table 1-6

References for SI citations

**Geological background of the Dengying Formation**

The Ediacaran succession in the Yangtze Gorges area is comprised of the Doushantuo and Dengying formations (Supplementary Fig. 2). The Doushantuo Formation is radiometrically dated between 635.2±0.6 Ma and 551.1±0.7 Ma^1^, while the Dengying Formation brackets the rest of Ediacaran Period (i.e. 551 to 541 Ma). In the Yangtze Gorges area, the Dengying Formation can be divided into three lithological members, in ascending order, the Hamajing, Shibantan, and Baimatuo members^2^. The Hamajing Member is characterized by light gray, medium- to thick-bedded dolostone with widespread tepee structures and karstification features^3,4^. The occurrences of ooids and oncolites grainstone indicate deposition at low sea level^3,5,6^. The overlying Shibantan Member is composed of dark gray, thin-bedded, laminated bituminous limestone. The lack of subaerial exposure structures and the occasional occurrences of hummocky cross-bedding structures suggests the deposition in deep subtidal environment and probably close to the storm wave base. The microlaminae are interpreted as microbial mats that trapped silts, clays, and micrites, and might have induced calcite precipitation^4,7^. The Baimatuo Member consists of light gray, thick-bedded dolostone. The Baimatuo Member is unconformably overlain by the Yanjiahe Formation which contains basal Cambrian small shelly fossils and acritarchs^8-10^.

The Shibantan member is one of a few well-preserved terminal Ediacaran fossiliferous carbonate sequences in the world. It contains abundant microbial structures and macroscopic fossils, including the algal fossil *Vendotaenia antiqua*^11-13^, Ediacara-type fossils *Paracharnia dengyingensis*^11,14^, *Yangtziramulus Zhangi*^3,15^, *Pteridinium*, *Rangea*, *Charniodiscus*, *Hiemalora*^16^, annulated tubular fossil *Wutubus annularis*^16^, and possible sponge spicules^2,17^. In addition, abundant trace fossils were discovered on bedding surface covered with microbial structures such as elephant-skin like structures^4,7,18,19^. Recent discovery of the oldest trackways suggests the occurrences of bilaterian animals with paired appendages ^20^.

The rock samples in this study were collected from the Shibantan Member at the Sixi and Huangniuyan sections in the Yangtze Gorges area. 32 samples from the Sixi Section and 100 samples from the Huangniuyan Section were analyzed (Supplementary Fig. 1).

**Geological background of carbonates in Late Paleozoic**

The late Devonian to early Carboniferous Paleogeography in South China was characterized by the development of isolated carbonate platforms that were separated by intraplatform basins^21,22^. The platform succession is dominated by thick-bedded skeletal wackstone, packstone, and grainstone with occasional occurrences of oolitic grainstone, while the basinal sections are mainly composed of nodular limestone or thin bedded lime mudstone and wackstone^23^. Abundant benthic fossils, including brachiopods, corals, and echinoderms, are discovered from both shelf and basinal sections^24,25^. Totally 6 sections were sampled, and a brief description of the studied sections are displayed below (Supplementary Fig. 3).

In the Panlong section (Guangxi Province), the late Devonian Rongxian Formation mainly consists of bioclastic wackstone/packstone and oolitic grainstone, representing the shallow marine carbonate platform deposition. It yields abundant benthic fossils, including brachiopods, corals, ostracods, stromatoporoids and bryozoans. The successions in the Madao and Dazhai sections in Guizhou Province, aged from Late Devonian to Early Carboniferous, can be partially correlated with the Rongxian Formation in the Panlong Section, and are mainly composted of massive shallow water bioclastic wackstone/packstone/grainstone. We sampled the micritic limestone with little fossil records and bioturbation signature.

The deep water carbonate samples were collected from the Wuzhishan formations in the Duli, Xiada, and Daposhang sections in Guizhou provinces. These sections were deposited in the intraplatform basin environments. The Wuzhishan Formation is mainly composed of thin bedded limestone and nodular lime mudstone and wackstone, with occasional occurrences of packstone, and can be correlated to the Rongxian Formation in the carbonate platform environment. Abundant benthic fossils such as brachiopods and echinoderms were discovered from these sections. We sampled the micritic limestone with little fossil records and bioturbation signature.

**Fe_carb_ as a new proxy for redox conditions.**

As is stated in the main text, when pH and temperature remain unchanged, there is an exponential relationship between benthic Fe^2+^ flux (Flux_Fe_) which could eventually reach the seafloor and bottom water oxygen (O_2-BW_), which can be expressed as Eq. 8:

|  | ${Flux}_{Fe}=a\times\left( O_{2-BW} \right)^{b}$ | **[8]** |
| --- | --- | --- |

Taking the logarithm of both sides gives:

|  | $\log\left( {Flux}_{Fe} \right)=\log\left( a\times\left( O_{2-BW} \right)^{b} \right)$ | **[S1]** |
| --- | --- | --- |
|  | $\log\left( {Flux}_{Fe} \right)=\log\left( a \right)+b\times log(O_{2-BW})$ | **[S2]** |

Eq. **S2** indicates that there is a linear relationship between log(Flux_Fe_) and log(O_2-BW_). Previous studies have reported the data of benthic Fe^2+^ flux on the seafloor and bottom oxygen using different analytical methodologies^26-34^. In this study, instead of using in situ fluxes, we collected the benthic flux data measured by non-invasive benthic chambers^35^. Benthic Fe^2+^ flux and O_2-BW_ are measured simultaneously from the Californian coast, shelf and slope^26,27,34^. We selected data from the locations with water depth greater than 500 m, where potential hydrodynamic influence and bioturbation could be minimum. Least square method is applied to fit the linear Eq. **S2** between Flux_Fe_ and O_2-BW_ (Supplementary Fig. 7). Coefficients a and b with one standard error are presented below:

|  | $a={10}^{-4.98\pm0.72}$ | **[S3]** |
| --- | --- | --- |
|  | $b=-1.71\pm0.16$ | **[S4]** |
|  | $R^{2}=0.6271$ | **[S5]** |

Thus Eq. **8** can be rewritten as Eq. **9**:

|  | ${Flux}_{Fe}={10}^{-4.98\pm0.72}\times\left( O_{2-BW} \right)^{-1.71\pm0.16}$ | **[9]** |
| --- | --- | --- |

In Eq. **9**, the units of Flux_Fe_ and O_2-BW_ are mol·m^-2^·Myr^-1^ and mol·L^-1^. The standard errors for coefficients a and b can be used in sensitivity tests hereinafter.

During carbonate precipitation on the seafloor, seawater Fe^2+^ seawater and benthic Fe^2+^ flux can both incorporate into the carbonate lattice as Fe_carb_. However, Fe^2+^ content in seawater (0~1.5 nmol·L^-1^ in modern ocean) is much less than that in Fe^2+^ supply from porewater (0~500 μmol·L^-1^ in modern ocean), i.e., Flux_Fe_ (0.02~568 μmol·m^-2^·d^-1^ in modern ocean)^26,27,34^. Therefore, Flux_Fe_ on the seafloor could be the main contributor to the Fe_carb_, and the correlation between the Flux_Fe_ and Fe_carb_ is expressed by the following equation:

|  | $K_{Fe}\times{Flux}_{Fe}\times t\times A\times M_{Fe}=A\times s\times t\times\rho\times Fe_{carb}$ | **[S6]** |
| --- | --- | --- |

where $K_{\mathrm{Fe}}$ is the partitioning coefficient for the benthic Fe^2+^ flux into the carbonate lattice. t, A, s, $\rho$ are the time, area, sedimentation rate, and density of carbonates, respectively; M_Fe_ is the molecular weight of Fe. So, using the best fitted values (a=${10}^{-4.98}$, b=-1.71) and rearranging Eqs. **9** and **S6**, Fe_carb_ could be expressed as:

|  | ${Fe}_{carb}=\frac{K_{Fe}\times M_{Fe}\times{10}^{-4.98}\times\left( O_{2-BW} \right)^{-1.71}}{s\times\rho}$ | **[10]** |
| --- | --- | --- |

where M_Fe_ = 56 g/mol and ρ = 2.71×10^6^ g/m^3^. Eq. **10** indicates that Fe_carb_ is inversely correlated with both bottom oxygen (O_2-BW_) and sedimentation rate (s). To modeling the Eq. **10**, we use the following sedimentation rates: s=5, 10, 20, 40 m/Myr.

Fe_carb_ in this model mainly depends on the partitioning coefficient for the benthic Fe^2+^ flux incorporated into the carbonate lattice (K_Fe_). Nonetheless, tightly constrained due to rare Fe_carb_ data of the modern limestone. Although some experimental work has been done regarding Fe^2+^ incorporation into calcite ^36,37^, K_Fe_ still cannot be tightly constrained in our case when the concentration of benthic Fe^2+^ flux is not in equilibrium with the aqueous solution surrounding the calcite. Alternatively, we use Fe_carb_ of the late Devonian-early Carboniferous shallow marine carbonates from the Madao, Panlong and Dazhai sections in South China (see supplementary text) to calculate the K_Fe_ by transformation of Eq. **10**:

|  | $K_{Fe}=\frac{Fe_{carb}\times s\times\rho}{M_{Fe}\times{10}^{-4.98}\times\left( O_{2-BW} \right)^{-1.71}}$ | **[S7]** |
| --- | --- | --- |

It is reasonable to speculate that the dissolved oxygen in late Devonian shallow seawater was approximately in equilibrium with the atmosphere, whose *p*O_2_ level was at least comparable to or even higher than that of the modern atmosphere^38,39^. Thus, late Paleozoic shallow-water carbonates may precipitate from well mixed and oxygenated seawater that is similar to present state. The atmospheric *p*O_2_ level at present is 0.21 atm. Assuming the seawater temperature is 25℃, we calculate the saturated O_2_ in seawater by Henry’s Law in Eq. **S8**:

|  | $O_{2-sw}=H\times O_{2-atm}$ | **[S8]** |
| --- | --- | --- |

where H is Henry’s law constant for O_2_ which equals to 1.3 mmol·L^-1^·atm^-1^. O_2-sw_ is the O_2_ concentration in seawater and O_2-atm_ is the atmospheric *p*O_2_ level. Therefore, the saturated O_2_ content in seawater is 273 μmol·L^-1^. Conservatively estimated O_2-BW_ of 250 μmol·L^-1^ during the late Devonian is used in the calculation. Assuming the sedimentation rates are 12.5 m/Myr, 6.4 m/Myr and 28.6 m/Myr for the Madao, Panlong and Dazhai sections, respectively (Supplementary Table 3), the calculated K_Fe_ is 1.86 for the Madao section, 1.89 for the Panlong section, and 3.22 for the Dazhai section.

To justify the K_Fe_ values, the seafloor O_2_ fugacity in the equivalent deep water sections are calculated by Eq. **S9**. The Fe_carb_ and s for the three deep water sections were set to the following values: the Duli section (Fe_carb_=1168.20 ppm, s=4.2 m/Myr), the Xiada section (Fe_carb_=545.27 ppm, s=4.2 m/Myr) and the Daposhang section (Fe_carb_=1080.96 ppm, s=5.6 m/Myr) (see supplementary text; Supplementary Table 2 & 3).

|  | $O_{2-BW}=\left( \frac{Fe_{carb}\times s\times\rho}{M_{Fe}\times{10}^{-4.98}\times K_{Fe}} \right)^{-\frac{1}{1.71}}$ | **[S9]** |
| --- | --- | --- |

Assigning 2.32 to K_Fe_ (the average value of the platform sections), the calculated O_2-BW_ values for the Duli, Xiada and Daposhang sections are 81.70 μmol/L, 127.57 μmol/L and 72.26 μmol/L, respectively. These values are above the threshold of oxic condition (>68 μmol/L)^40,41^, consistent with the presence of abundant benthic and sessile organisms in these deep water sections^22,24,42^. Therefore, after determining K_Fe_=2.32, we can use Fe_carb_ and sedimentation rate to calculate the bottom O_2_ level at WSI by Eq. **S9**.

We limit O_2-BW_ within the range of 6.25μmol/L (the redox boundary between the anoxic and euxinic condition) to 273μmol/L (water with saturated O_2_ at present by Henry’s Law). Oxic (>68μmol/L), suboxic (13.6μmol/L~68μmol/L) and anoxic conditions (6.25μmol/L ~13.6μmol/L) are shown in the modeling results (Fig. 4)^40,41^.

**Sensitivity tests for the modeling.**

**a) Fitting coefficients in Eq. 9.** The confidence intervals of coefficients a and b can constrain the best fitting line by the limit lines as follows:

|  | ${Flux}_{Fe}={10}^{-5.70}\times\left( O_{2-BW} \right)^{-1.87}$ | **[S10]** |
| --- | --- | --- |
|  | ${Flux}_{Fe}={10}^{-4.26}\times\left( O_{2-BW} \right)^{-1.55}$ | **[S11]** |

The best fitting line can be expressed as:

|  | ${Flux}_{Fe}={10}^{-4.98}\times\left( O_{2-BW} \right)^{-1.71}$ | **[S12]** |
| --- | --- | --- |

It turns out the three curves represented by Eqs. **S10-S12** intersect at one point (O_2-BW_=31.62 μmol·L^-1^, Flux_Fe_=1.42 μmol·m^-2^·d^-1^) with little deviation in the overall trend and calculated values (Supplementary Fig. 8a). When O_2-BW_ is 6.25μmol·L^-1^, the best fitted value of benthic Fe^2+^ flux is 22.74μmol·m^-2^·d^-1^ and the interval of possible benthic Fe^2+^ flux values is [17.54μmol·m^-2^·d^-1^, 29.47μmol·m^-2^·d^-1^]; when O_2-BW_ is 273μmol·L^-1^, the best fitted value of benthic Fe^2+^ flux is 0.036μmol·m^-2^·d^-1^ and the interval of possible benthic Fe^2+^ flux values is [0.025μmol·m^-2^·d^-1^, 0.051μmol·m^-2^·d^-1^]. As the bottom oxygen level decreases, the predicted flux’s deviation from the best fitted value decreases first and then increases. We then calculate the Fe_carb_ change according to different fitting coefficients by Eq. **10** with the same sedimentation rate of 20 m/Myr (similar trend when applied to different sedimentation rates). Still, there is little change on the trend and magnitude of modeling results (Supplementary Fig. 8b). When the O_2-BW_ is 6.25μmol·L^-1^, the best fitted value of Fe_carb_ is 1.99×10^4^ppm and the interval of possible Fe_carb_ values is [1.53×10^4^ppm, 2.58×10^4^ppm]; when the O_2-BW_ is 273μmol·L^-1^, the best fitted value of Fe_carb_ is 31.32ppm and the interval of possible Fe_carb_ values is [22.20ppm, 44.21ppm]. Furthermore, in the other scenario of the late Devonian deep water carbonates, we also assign a series of values to the coefficients and calculate O_2-BW_ by Eq. **10**. When a=10^-5.70^ and b=-1.87, the O_2-BW_ values of the Duli, Xiada, and Daposhang sections are 75.33 μmol·L^-1^, 113.22 μmol·L^-1^ and 67.33 μmol·L^-1^, respectively; when a=10^-4.26^ and b=-1.55, the O_2-BW_ values of the Duli, Xiada, and Daposhang sections are 90.12 μmol·L^-1^, 147.33 μmol·L^-1^ , and 78.69 μmol·L^-1^, respectively All the results indicate an oxic condition despite of fluctuating assignment. Therefore, the fitting coefficients in Eq. **9** have limited impact on the modeling result.

**b) Partitioning coefficient of the benthic iron flux into the carbonate lattice (K_Fe_).** As discussed above, there is uncertainty on the exact value of K_Fe_ due to the lack of modern carbonate Fe_carb_ with different bottom oxygen levels. Despite the fact that we use the late Devonian carbonate Fe_carb_ for the calculation, we still could not constrain the bottom O_2_ level in the shallow water during that period. The O_2_-saturated water contains 273 μmol·L^-1^ of oxygen according to Henry’s Law. Therefore, we explored the effect of a wide range of O_2-BW_ (200~273 μmol·L^-1^) of the late Devonian shallow platform. O_2-BW_ can affect the value of K_Fe_, which ultimately impinge the calculation. K_Fe_ ranges from 2.23 to 3.80 for the Dazhai section, from 1.27 to 2.16 for the Madao section and from 1.28 to 2.19for the Panlong section. The average of K_Fe_ values varies from 1.60 to 2.72. The results of calculation by Eq. **S7** are summarized in Supplementary Fig. 8c, indicating small variation of K_Fe_. Then we apply the adjusted K_Fe_ values to calculate Fe_carb_ with the same sedimentation rate of 20 m/Myr. The results show that there is still no significant difference on the trend or magnitude of the final modeling results (Supplementary Fig. 8d). When the O_2-BW_ is 6.25μmol·L^-1^, the interval of possible Fe_carb_ values is [1.37×10^4^ppm, 2.33×10^4^ppm]; when the O_2-BW_ is 273μmol·L^-1^, the interval of possible Fe_carb_ values is [21.56ppm, 36.71ppm]. Again, we use the average K_Fe_ to calculate the O_2-BW_ by Eq. **S10** for late Devonian deep water carbonates. When K_Fe_ is assigned by a series of values, ranging from 1.60 to 2.72, the ranges of the O_2-BW_ of the Duli, Xiada, and Daposhang sections are [65.75 μmol·L^-1^, 89.67 μmol·L^-1^], [102.66 μmol·L^-1^, 140.01 μmol·L^-1^] and [58.15 μmol·L^-1^, 79.30 μmol·L^-1^], respectively. Most O_2-BW_ data coincide with the range of oxic condition (>68 μmol/L). Therefore, the partitioning coefficient of the benthic Fe^2+^ flux into the carbonate lattice (K_Fe_) has minor effect on the simulations.


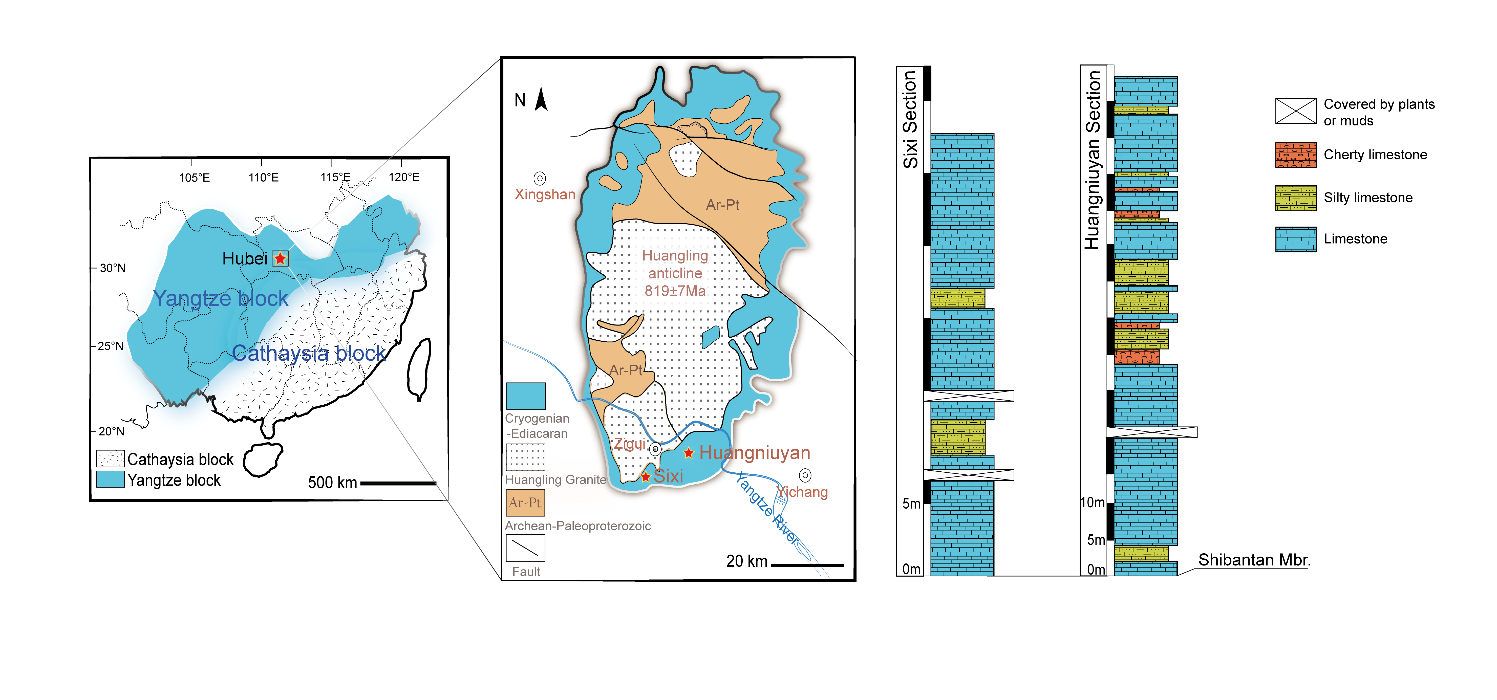
Supplementary Fig. 1. Simplified map showing the sampling localities (the Sixi and Huangniuyan Sections, red stars) and stratigraphic column of the lower part of the Shibantan Member, Dengying Formation at the Sixi and Huangniuyan sections, Hubei Province, South China.


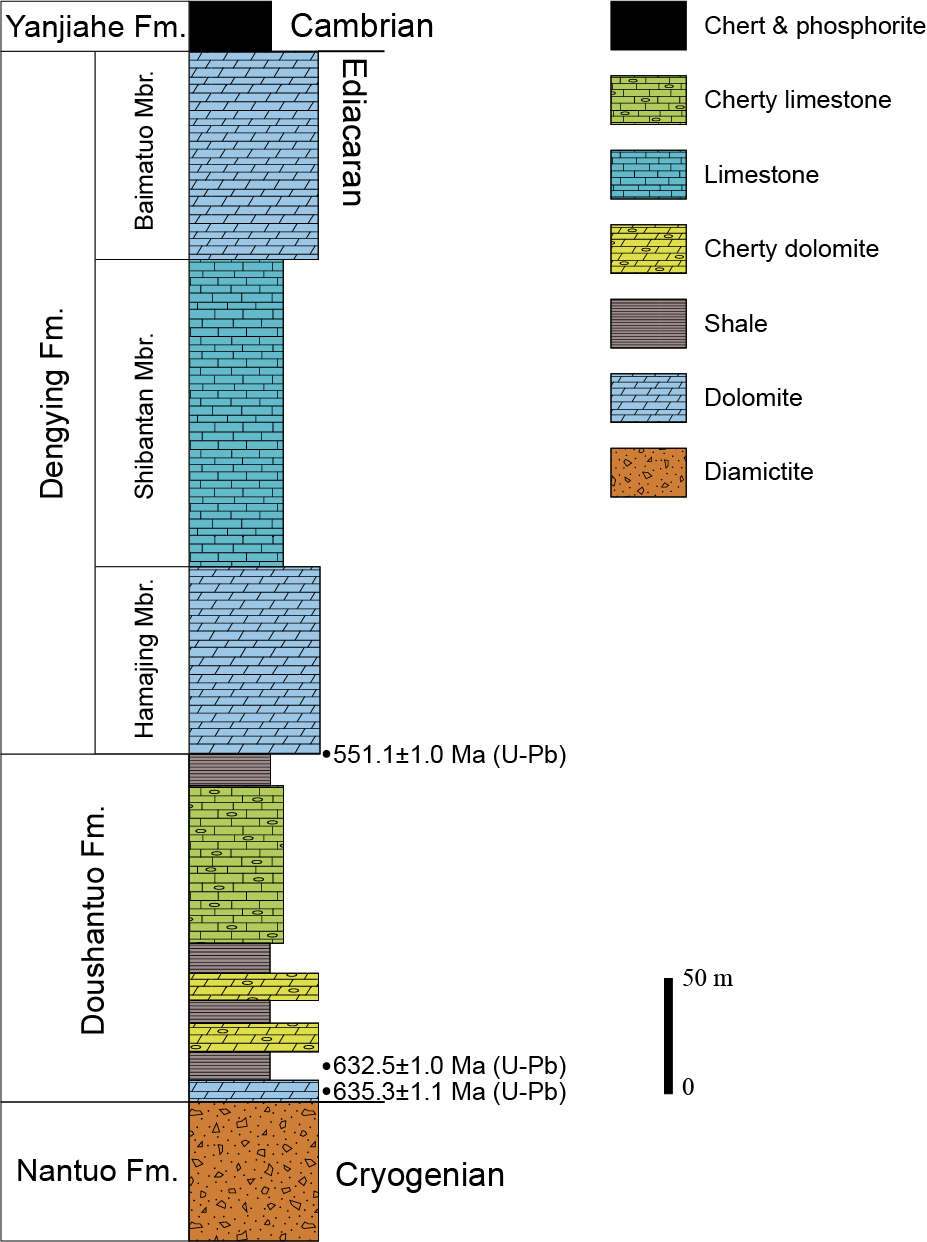
Supplementary Fig. 2. Simplified stratigraphic column together with chronology of Ediacaran Doushantuo and Dengying Formation and early Cambrian Yanjiahe Formation. Mbr.: Member; Fm.: Formation;


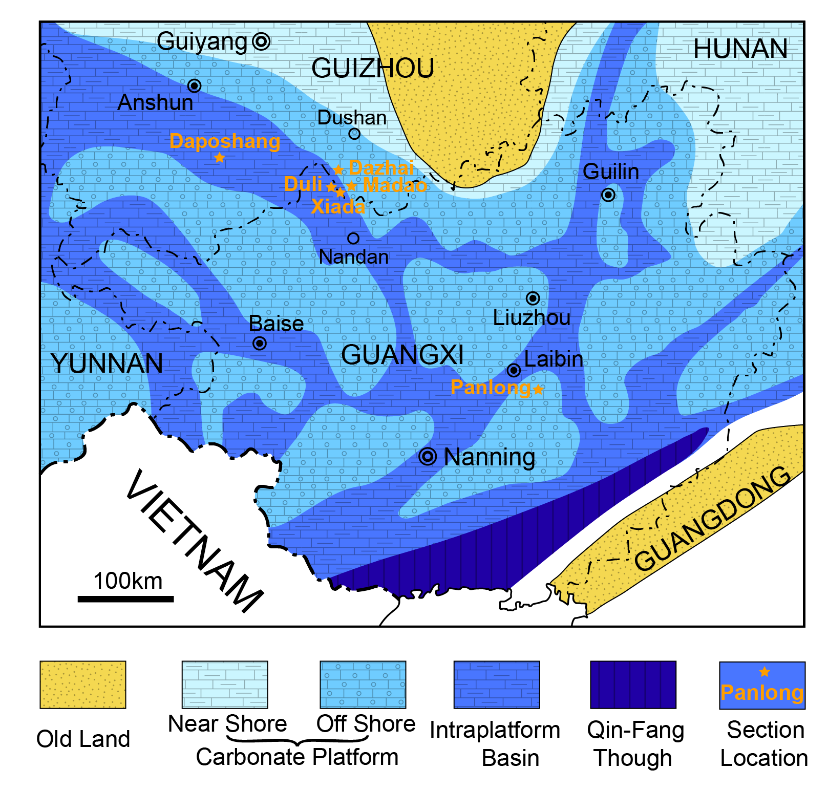
 Supplementary Fig. 3. Simplified map showing the sampling localities of the Late Paleozoic carbonates (orange stars) in the Cathysia Block. The Panlong, Madao and Dazhai sections are located on the shallow carbonate platform. Carbonate samples from Duli, Xiada and Daposhang Section deposited in deep water environment (intraplatform basin).

Supplementary Fig. 4. Fe_carb_ concentration of the Dengying Formation, all Samples included. Cross-plot shows Mg/Ca molar ratio(x-axis) vs. Fe_carb_ content(y-axis). Fe_carb_ content of both micrite and calcspar micro-laminae is plotted, showing no significant difference. Fe_carb_ concentration ranges from 2.27ppm to 260.15ppm.

Supplementary Fig. 5. a, Mg/Ca ratio of the Sixi section. b, Fe_carb_ of the Sixi section. c, Mg/Ca ratio of the Huangniuyan section. d, Fe_carb_ of the Huangniuyan section.

Supplementary Fig. 6. Benthic iron flux detected in the modern ocean. Benthic iron flux with depositional depth greater than 500m are systematically lower than those with depositional depth less than 250m. The data of benthic iron flux with depositional depth between 250m and 500m is scarce. The literatures referred to are listed^26,27,29,34^.


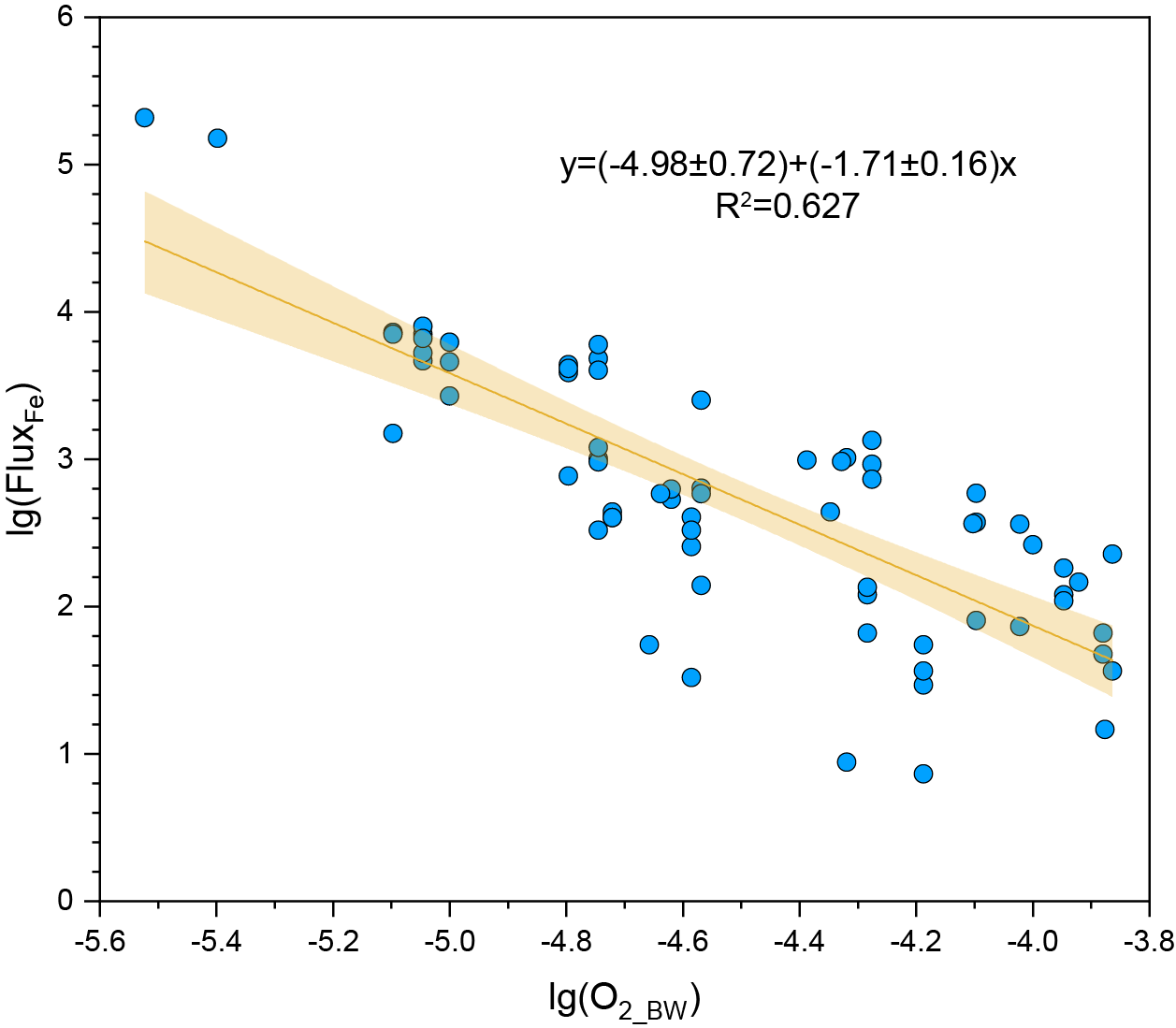
Supplementary Fig. 7. lg(Flux_Fe_) varies as a linear function of lg(O_2-BW_). The unit of Flux_Fe_ reaching on the seafloor is mol·m^-2^·Myr^-1^ and the unit of O_2-BW_ is mol·L^-1^. The fitting line with its 95% confidence interval controlled by the standard errors of intercept and slope are shown here.


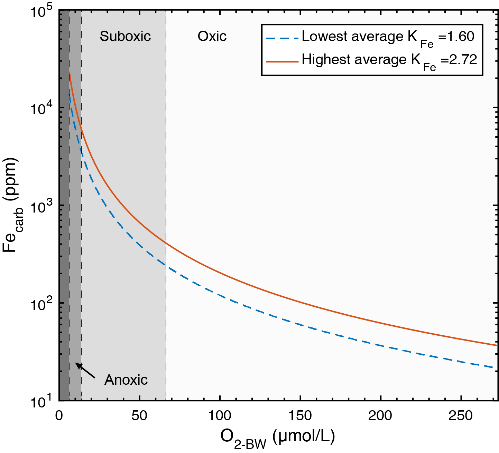

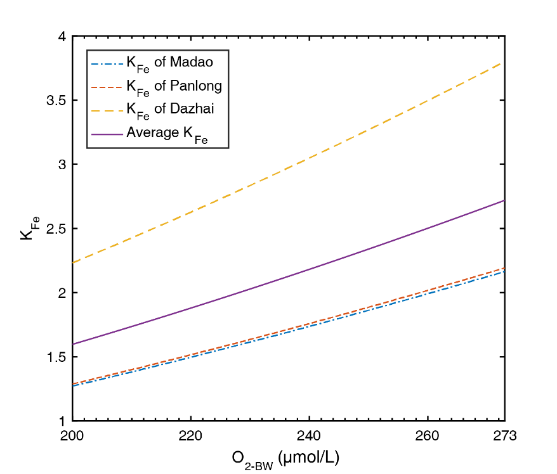

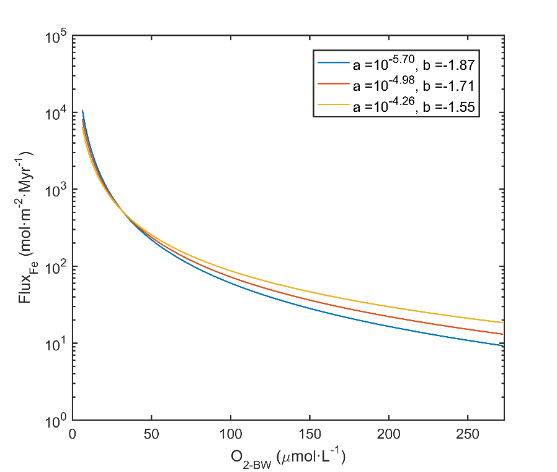

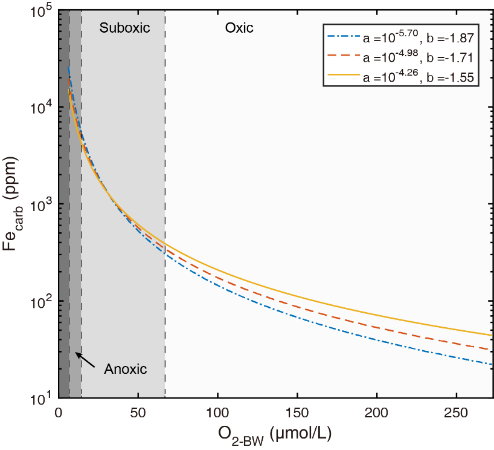
Supplementary Fig. 8. a, Sensitivity test for the coefficients’ standard errors of Eq. 9. b, Sensitivity test for the impact of parameters in Eq. 9 on the final Fe_carb_ results. The sedimentation rate is set to 20m/Myr. c, Sensitivity test for the impact of speculated O_2-BW_ on the parameter K_Fe_ by Eq. S7 during the deposition of Late Devonian shallow water carbonates. d, Sensitivity test for the impact of parameter K_Fe_ in Eq. S7 on the final Fe_carb_ results. The sedimentation rate is set to 20m/Myr.

d

c

a

b


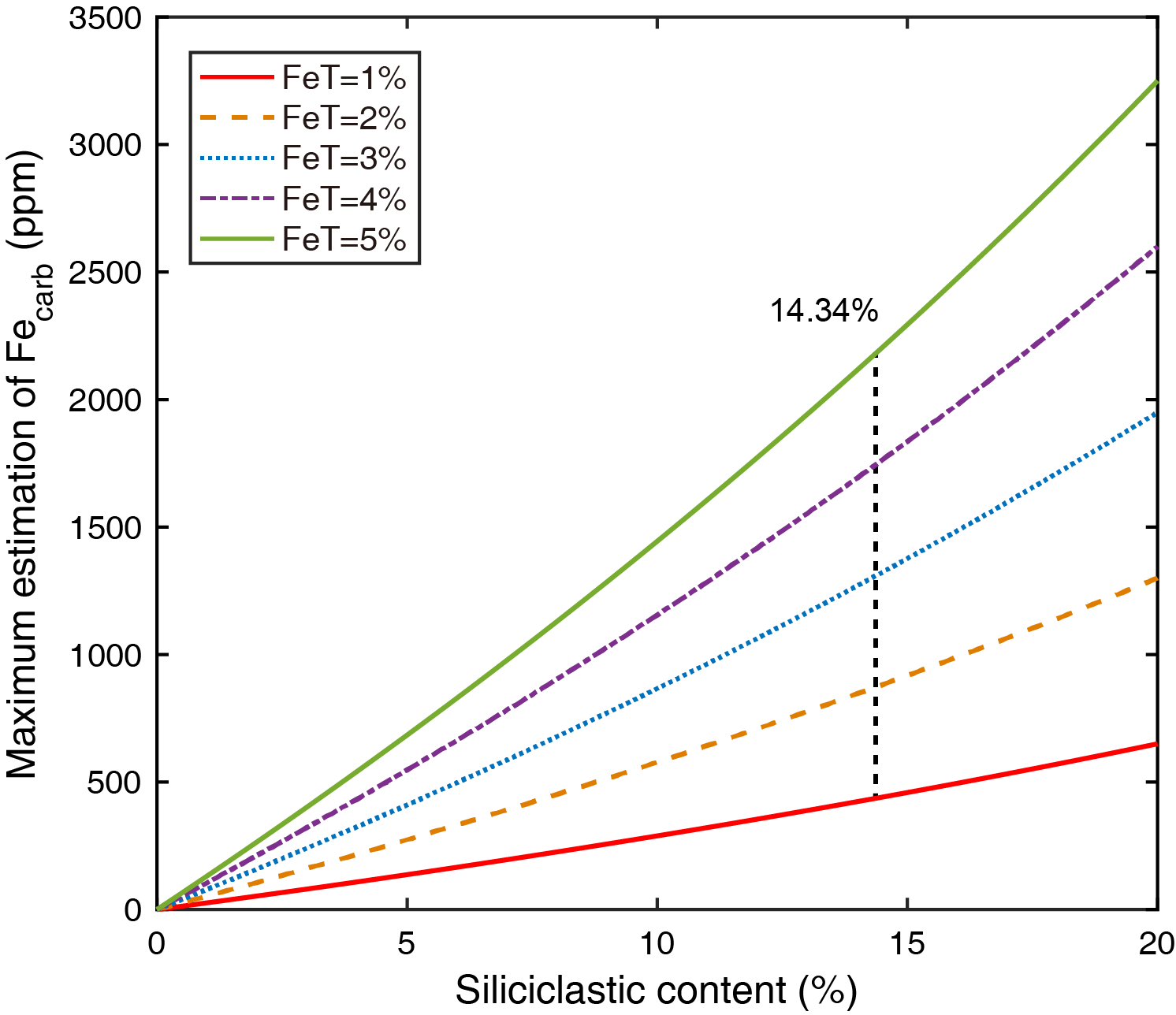
Supplementary Fig. 9. Maximum of Fe_carb_ provided by highly reactive Fe (Fe_HR_) in siliciclastic content. Cross-plot shows siliciclastic component (x-axis) vs. maximum estimation of Fe_carb_ content (y-axis). Fe_carb_ is controlled by the supply of Fe_HR_ in sediments. Considering the ratio of total iron content (Fe_T_, 1%-5%) and ratio of Fe_HR_ and Fe_T_ (Fe_HR_/Fe_T_, 0.26) ^43^, we can calculate the maximum estimation of Fe_carb_ provided by remaining Fe_HR_ if Fe_HR_ is totally transformed into Fe_carb_ by DIR. Dengying limestone has an average siliciclastic content of 14.34%, which correspond to maximum Fe_carb_ of at least 435 ppm (when Fe_T_=1%). Therefore, reactive Fe is sufficient when Fe_carb_ is used to reconstruct the seafloor redox when Dengying Formation deposited.

Supplementary Table 1. Major elements of the Dengying Formation, Hubei Province, South China. n. d. : not detected.

| Sample | Lithology | Mg/Ca | Fe_carb_ (ppm) | Mn_carb_ (ppm) |
| --- | --- | --- | --- | --- |
| SX-13 | micrite | 0.03 | 52.15 | n. d. |
| SX-15-02 | micrite | 0.02 | 7.27 | n. d. |
| SX-17-02 | micrite | 0.01 | 47.24 | n. d. |
| SX-20-03 | micrite | 0.07 | 87.88 | n. d. |
| SX-23 | micrite | 0.43 | 109.86 | 13.45 |
| SX-27-01 | micrite | 0.17 | 102.67 | 14.00 |
| SX-28-01 | micrite | 0.02 | 25.15 | n. d. |
| SX-30-02 | micrite | 0.22 | 63.41 | 12.27 |
| SX-31-01 | micrite | 0.11 | 44.91 | 6.42 |
| HNY-53-02 | micrite | 0.16 | 28.50 | 39.47 |
| HNY-54-02 | micrite | 0.01 | 23.36 | 14.02 |
| HNY-55-02 | micrite | 0.01 | 16.11 | 92.07 |
| HNY-56-02 | micrite | 0.46 | 157.34 | 0.00 |
| HNY-59-02 | micrite | 0.18 | 27.56 | n. d. |
| HNY-72-02 | micrite | 0.29 | 30.32 | n. d. |
| HNY-75-02 | micrite | 0.07 | 15.24 | n. d. |
| HNY-80-01 | micrite | 0.03 | 56.10 | n. d. |
| HNY-85-01 | micrite | 0.05 | 20.70 | n. d. |
| HNY-92-02 | micrite | 0.04 | 2.27 | n. d. |
| HNY-96-02 | micrite | 0.46 | 87.68 | 6.12 |
| HNY-98-02 | micrite | 0.11 | 18.38 | 6.89 |
| HNY-100-02 | micrite | 0.21 | 20.71 | 9.20 |
| SX-15-01 | calcspar | 0.05 | 56.37 | n. d. |
| SX-17-01 | calcspar | 0.14 | 160.26 | 0.00 |
| SX-20-02 | calcspar | 0.24 | 260.15 | 11.51 |
| SX-25 | calcspar | 0.08 | 38.15 | n. d. |
| SX-27-02 | calcspar | 0.05 | 233.63 | 0.00 |
| SX-28-02 | calcspar | 0.08 | 180.58 | 8.81 |
| SX-30-01 | calcspar | 0.01 | 9.11 | n. d. |
| SX-31-02 | calcspar | 0.03 | 26.12 | 0.00 |
| SX-32 | calcspar | 0.02 | 24.88 | 15.83 |
| HNY-51 | calcspar | 0.06 | 38.24 | 20.25 |
| HNY-53-01 | calcspar | 0.03 | 8.95 | 6.71 |
| HNY-54-01 | calcspar | 0.01 | 16.21 | 0.00 |
| HNY-55-01 | calcspar | 0.01 | 9.13 | 2.28 |
| HNY-56-01 | calcspar | 0.20 | 105.59 | n. d. |
| HNY-59-01 | calcspar | 0.04 | 26.77 | n. d. |
| HNY-62 | calcspar | 0.06 | 33.29 | 0.00 |
| HNY-65 | calcspar | 0.04 | 8.88 | n. d. |
| HNY-67 | calcspar | 0.04 | 6.75 | n. d. |
| HNY-69-01 | calcspar | 0.12 | 109.04 | 0.00 |
| HNY-72-01 | calcspar | 0.15 | 80.04 | n. d. |
| HNY-75-01 | calcspar | 0.24 | 28.42 | n. d. |
| HNY-78 | calcspar | 0.02 | 85.43 | n. d. |
| HNY-80-02 | calcspar | 0.02 | 7.08 | n. d. |
| HNY-82 | calcspar | 0.23 | 51.52 | 2.06 |
| HNY-85-02 | calcspar | 0.04 | 13.72 | n. d. |
| HNY-88 | calcspar | 0.04 | 55.64 | n. d. |
| HNY-92-01 | calcspar | 0.01 | 2.31 | n. d. |
| HNY-94 | calcspar | 0.06 | 89.91 | n. d. |
| HNY-96-01 | calcspar | 0.30 | 10.35 | 0.00 |
| HNY-98-01 | calcspar | 0.02 | 4.41 | n. d. |
| HNY-99 | calcspar | 0.05 | 42.31 | n. d. |
| HNY-100-01 | calcspar | 0.02 | 4.52 | 4.52 |

Supplementary Table 2. Fe_carb_ of carbonate in the late Paleozoic. Fm. is the abbreviation of Formation.

| Section | Sample | Geological Unit | Age (Ma) | Fe_carb_ (ppm) | Mg/Ca | Lithology | Facies | Reference |
| --- | --- | --- | --- | --- | --- | --- | --- | --- |
| Panlong | NPL-22 | Rongxian Fm. | 373-372.2 | 86.02 | 0.01 | Limestone | Open platform | This study |
|  | NPL-24 | Rongxian Fm. | 373-372.2 | 77.85 | 0.03 | Limestone | Open platform | This study |
|  | NPL-31 | Rongxian Fm. | 373-372.2 | 51.82 | 0.02 | Limestone | Open platform | This study |
|  | NPL-37 | Rongxian Fm. | 373-372.2 | 69.81 | 0.01 | Limestone | Open platform | This study |
|  | NPL-43 | Rongxian Fm. | 373-372.2 | 67.37 | 0.01 | Limestone | Open platform | This study |
|  | NPL-48 | Rongxian Fm. | 373-372.2 | 74.08 | 0.01 | Limestone | Open platform | This study |
|  | NPL-51 | Rongxian Fm. | 373-372.2 | 166.26 | 0.01 | Limestone | Open platform | This study |
|  | NPL-54 | Rongxian Fm. | <372.2 | 86.29 | 0.01 | Limestone | Open platform | This study |
|  | NPL-4-1 | Rongxian Fm. | 372.2-371.5 | 95.09 | 0.01 | Limestone | Open platform | This study |
|  | NPL-4-3 | Rongxian Fm. | 372.2-371.5 | 112.68 | 0.01 | Limestone | Open platform | This study |
|  | NPL-4-5 | Rongxian Fm. | 372.2-371.5 | 97.43 | 0.01 | Limestone | Open platform | This study |
|  | NPL-4-11 | Rongxian Fm. | 372.2-371.5 | 95.83 | 0.01 | Limestone | Open platform | This study |
|  | NPL-4-19 | Rongxian Fm. | 372.2-371.5 | 100.89 | 0.01 | Limestone | Open platform | This study |
|  | NPL-4-23 | Rongxian Fm. | 371 | 107.64 | 0.01 | Limestone | Open platform | This study |
| Dazhai | DtZ1-(-3) | Rongxian Fm. | 370.7-370 | 47.57 | 0.01 | Limestone | Offshore platform | This study |
|  | DtZ1-(-2) | Rongxian Fm. | 370.7-370 | 25.89 | 0.01 | Limestone | Offshore platform | This study |
|  | DtZ1-0 | Rongxian Fm. | 370.7-370 | 62.22 | 0.01 | Limestone | Offshore platform | This study |
|  | DtZ1-1 | Rongxian Fm. | 370.7-370 | 19.06 | 0.01 | Limestone | Offshore platform | This study |
|  | DtZ1-c-17 | Rongxian Fm. | 370.7-370 | 33.50 | 0.01 | Limestone | Offshore platform | This study |
|  | DtZ1-bu-1 | Rongxian Fm. | 370.7-370 | 15.28 | 0.01 | Limestone | Offshore platform | This study |
|  | DtZ3-4 | Rongxian Fm. | 370.7-370 | 65.18 | 0.01 | Limestone | Offshore platform | This study |
|  | DtZ3-5 | Rongxian Fm. | 370.7-370 | 29.61 | 0.01 | Limestone | Offshore platform | This study |
|  | DtZ3-6 | Rongxian Fm. | 370.7-370 | 32.14 | 0.01 | Limestone | Offshore platform | This study |
|  | DtZ3-7 | Rongxian Fm. | 370.7-370 | 21.24 | 0.01 | Limestone | Offshore platform | This study |
| Madao | MD-1 | Rongxian Fm. | 359 | 67.48 | 0.01 | Limestone | Offshore platform | This study |
|  | MD-8 | Rongxian Fm. | 359-358 | 58.92 | 0.01 | Limestone | Offshore platform | This study |
|  | MD-11 | Rongxian Fm. | 359-358 | 59.89 | 0.01 | Limestone | Offshore platform | This study |
|  | MD-16 | Rongxian Fm. | 357-355 | 31.41 | 0.01 | Limestone | Offshore platform | This study |
|  | MD-24 | Rongxian Fm. | 357-355 | 33.63 | 0.02 | Limestone | Offshore platform | This study |
|  | MD-30 | Rongxian Fm. | 357-355 | 25.50 | 0.01 | Limestone | Offshore platform | This study |
|  | MD-33 | Rongxian Fm. | 357-355 | 48.96 | 0.01 | Limestone | Offshore platform | This study |
| Duli | DuL10-1a | Wuzhishan Fm. | 359.3-359.1 | 625.38 | 0.01 | Limestone | Slope to intraplatform basin | This study |
|  | DuL10-1b | Wuzhishan Fm. | 359.3-359.1 | 484.44 | 0.01 | Limestone | Slope to intraplatform basin | This study |
|  | DuL10-2a | Wuzhishan Fm. | 359.3-359.1 | 1499.77 | 0.01 | Limestone | Slope to intraplatform basin | This study |
|  | DuL10-2b | Wuzhishan Fm. | 359.3-359.1 | 1794.19 | 0.01 | Limestone | Slope to intraplatform basin | This study |
|  | DuL10-2c | Wuzhishan Fm. | 359.3-359.1 | 981.45 | 0.01 | Limestone | Slope to intraplatform basin | This study |
|  | DuL-14a | Wuzhishan Fm. | 359.1-358.9 | 1652.83 | 0.01 | Limestone | Slope to intraplatform basin | This study |
|  | DuL-14b | Wuzhishan Fm. | 359.1-358.9 | 961.36 | 0.01 | Limestone | Slope to intraplatform basin | This study |
|  | DuL-15a | Wuzhishan Fm. | 358.9-358.4 | 383.03 | 0.01 | Limestone | Slope to intraplatform basin | This study |
|  | DuL-15b | Wuzhishan Fm. | 358.9-358.4 | 1204.72 | 0.01 | Limestone | Slope to intraplatform basin | This study |
|  | DuL-16a | Wuzhishan Fm. | 358.9-358.4 | 2037.93 | 0.02 | Limestone | Slope to intraplatform basin | This study |
|  | DuL-16b | Wuzhishan Fm. | 358.9-358.4 | 2471.23 | 0.02 | Limestone | Slope to intraplatform basin | This study |
|  | DuL-16c | Wuzhishan Fm. | 358.9-358.4 | 1447.18 | 0.01 | Limestone | Slope to intraplatform basin | This study |
|  | DuL-16d | Wuzhishan Fm. | 358.9-358.4 | 992.46 | 0.01 | Limestone | Slope to intraplatform basin | This study |
|  | DuL-17a | Wuzhishan Fm. | 358.4-357.5 | 1363.32 | 0.02 | Limestone | Slope to intraplatform basin | This study |
|  | DuL-17b | Wuzhishan Fm. | 358.4-357.5 | 1585.22 | 0.02 | Limestone | Slope to intraplatform basin | This study |
|  | DuL-17c | Wuzhishan Fm. | 358.4-357.5 | 1035.07 | 0.01 | Limestone | Slope to intraplatform basin | This study |
|  | DuL-18a | Wuzhishan Fm. | 358.4-357.5 | 934.02 | 0.01 | Limestone | Slope to intraplatform basin | This study |
|  | DuL-18b | Wuzhishan Fm. | 358.4-357.5 | 627.24 | 0.01 | Limestone | Slope to intraplatform basin | This study |
|  | DuL-18c | Wuzhishan Fm. | 358.4-357.5 | 1671.64 | 0.01 | Limestone | Slope to intraplatform basin | This study |
|  | DuL-19a | Wuzhishan Fm. | 358.4-357.5 | 716.47 | 0.01 | Limestone | Slope to intraplatform basin | This study |
|  | DuL-19b | Wuzhishan Fm. | 358.4-357.5 | 682.48 | 0.01 | Limestone | Slope to intraplatform basin | This study |
|  | DuL-20a | Wuzhishan Fm. | 358.4-357.5 | 739.94 | 0.01 | Limestone | Slope to intraplatform basin | This study |
|  | DuL-20b | Wuzhishan Fm. | 358.4-357.5 | 977.34 | 0.01 | Limestone | Slope to intraplatform basin | This study |
| Xiada | xd12-20-1-1 | Wuzhishan Fm. | 359.3-359.1 | 594.02 | 0.01 | Limestone | Slope to intraplatform basin | This study |
|  | xd12-20-1-2 | Wuzhishan Fm. | 359.3-359.1 | 583.18 | 0.01 | Limestone | Slope to intraplatform basin | This study |
|  | xd12-20-2-1 | Wuzhishan Fm. | 359.3-359.1 | 449.71 | 0.01 | Limestone | Slope to intraplatform basin | This study |
|  | xd12-20-2-2 | Wuzhishan Fm. | 359.3-359.1 | 620.71 | 0.01 | Limestone | Slope to intraplatform basin | This study |
|  | xd12-21-1-1 | Wuzhishan Fm. | 359.3-359.1 | 605.35 | 0.01 | Limestone | Slope to intraplatform basin | This study |
|  | xd12-21-1-2 | Wuzhishan Fm. | 359.3-359.1 | 442.35 | 0.01 | Limestone | Slope to intraplatform basin | This study |
|  | xd12-21-2-1 | Wuzhishan Fm. | 359.3-359.1 | 579.30 | 0.01 | Limestone | Slope to intraplatform basin | This study |
|  | xd12-21-2-2 | Wuzhishan Fm. | 359.3-359.1 | 746.45 | 0.01 | Limestone | Slope to intraplatform basin | This study |
|  | xd12-22-1-1 | Wuzhishan Fm. | 359.3-359.1 | 423.84 | 0.01 | Limestone | Slope to intraplatform basin | This study |
|  | xd12-22-1-2 | Wuzhishan Fm. | 359.3-359.1 | 390.16 | 0.01 | Limestone | Slope to intraplatform basin | This study |
|  | xd12-22-2-1 | Wuzhishan Fm. | 359.3-359.1 | 474.22 | 0.01 | Limestone | Slope to intraplatform basin | This study |
|  | xd12-22-2-2 | Wuzhishan Fm. | 359.3-359.1 | 761.71 | 0.01 | Limestone | Slope to intraplatform basin | This study |
|  | xd12-23-1-1 | Wuzhishan Fm. | 359.3-359.1 | 616.66 | 0.01 | Limestone | Slope to intraplatform basin | This study |
|  | xd12-23-1-2 | Wuzhishan Fm. | 359.3-359.1 | 373.01 | 0.01 | Limestone | Slope to intraplatform basin | This study |
|  | xd12-23-2-1 | Wuzhishan Fm. | 359.3-359.1 | 448.45 | 0.01 | Limestone | Slope to intraplatform basin | This study |
|  | xd12-23-2-2 | Wuzhishan Fm. | 359.3-359.1 | 498.24 | 0.01 | Limestone | Slope to intraplatform basin | This study |
|  | xd12-24-1-1 | Wuzhishan Fm. | 359.3-359.1 | 555.77 | 0.01 | Limestone | Slope to intraplatform basin | This study |
|  | xd12-24-1-2 | Wuzhishan Fm. | 359.3-359.1 | 529.65 | 0.01 | Limestone | Slope to intraplatform basin | This study |
|  | xd12-24-2-1 | Wuzhishan Fm. | 359.3-359.1 | 511.05 | 0.01 | Limestone | Slope to intraplatform basin | This study |
|  | xd12-24-2-2 | Wuzhishan Fm. | 359.3-359.1 | 449.91 | 0.01 | Limestone | Slope to intraplatform basin | This study |
|  | xd12-25-1-1 | Wuzhishan Fm. | 359.3-359.1 | 446.03 | 0.01 | Limestone | Slope to intraplatform basin | This study |
|  | xd12-25-1-2 | Wuzhishan Fm. | 359.3-359.1 | 604.34 | 0.01 | Limestone | Slope to intraplatform basin | This study |
|  | xd12-25-2-1 | Wuzhishan Fm. | 359.3-359.1 | 522.46 | 0.01 | Limestone | Slope to intraplatform basin | This study |
|  | xd12-25-2-2 | Wuzhishan Fm. | 359.3-359.1 | 586.97 | 0.01 | Limestone | Slope to intraplatform basin | This study |
|  | xd12-26L-1 | Wuzhishan Fm. | 359.3-359.1 | 789.54 | 0.02 | Limestone | Slope to intraplatform basin | This study |
|  | xd12-26L-2 | Wuzhishan Fm. | 359.3-359.1 | 580.28 | 0.01 | Limestone | Slope to intraplatform basin | This study |
|  | xd12-26U-1 | Wuzhishan Fm. | 359.3-359.1 | 477.87 | 0.01 | Limestone | Slope to intraplatform basin | This study |
|  | xd12-26U-2 | Wuzhishan Fm. | 359.3-359.1 | 606.39 | 0.01 | Limestone | Slope to intraplatform basin | This study |
| Daposhang | 16dps-10-1 | Wuzhishan Fm. | 358 | 905.82 | 0.01 | Limestone | Slope to intraplatform basin | This study |
|  | 16dps-10-2 | Wuzhishan Fm. | 358 | 1006.30 | 0.02 | Limestone | Slope to intraplatform basin | This study |
|  | 16dps-11-1 | Wuzhishan Fm. | 358.9-358.4 | 1183.38 | 0.02 | Limestone | Slope to intraplatform basin | This study |
|  | 16dps-11-2 | Wuzhishan Fm. | 358.9-358.4 | 1209.69 | 0.02 | Limestone | Slope to intraplatform basin | This study |
|  | 16dps-12-1 | Wuzhishan Fm. | 358.9-358.4 | 2335.54 | 0.02 | Limestone | Slope to intraplatform basin | This study |
|  | 16dps-12-2 | Wuzhishan Fm. | 358.9-358.4 | 1569.62 | 0.02 | Limestone | Slope to intraplatform basin | This study |
|  | 16dps-13-1 | Wuzhishan Fm. | 358.9-358.4 | 1257.79 | 0.01 | Limestone | Slope to intraplatform basin | This study |
|  | 16dps-13-2 | Wuzhishan Fm. | 358.9-358.4 | 2010.06 | 0.01 | Limestone | Slope to intraplatform basin | This study |
|  | 16dps-14-1 | Wuzhishan Fm. | 358.9-358.4 | 1184.56 | 0.02 | Limestone | Slope to intraplatform basin | This study |
|  | 16dps-14-2 | Wuzhishan Fm. | 358.9-358.4 | 1092.57 | 0.01 | Limestone | Slope to intraplatform basin | This study |
|  | 16dps-15-1 | Wuzhishan Fm. | 358.9-358.4 | 1155.40 | 0.01 | Limestone | Slope to intraplatform basin | This study |
|  | 16dps-15-2 | Wuzhishan Fm. | 358.9-358.4 | 1169.92 | 0.01 | Limestone | Slope to intraplatform basin | This study |
|  | 16dps-16-1 | Wuzhishan Fm. | 358.9-358.4 | 874.26 | 0.01 | Limestone | Slope to intraplatform basin | This study |
|  | 16dps-16-2 | Wuzhishan Fm. | 358.9-358.4 | 915.05 | 0.01 | Limestone | Slope to intraplatform basin | This study |
|  | 16dps-17-1 | Wuzhishan Fm. | 358.9-358.4 | 1059.23 | 0.02 | Limestone | Slope to intraplatform basin | This study |
|  | 16dps-17-2 | Wuzhishan Fm. | 358.9-358.4 | 797.00 | 0.02 | Limestone | Slope to intraplatform basin | This study |
|  | 16dps-18-1 | Wuzhishan Fm. | 358.9-358.4 | 968.43 | 0.02 | Limestone | Slope to intraplatform basin | This study |
|  | 16dps-18-2 | Wuzhishan Fm. | 358.9-358.4 | 1001.86 | 0.02 | Limestone | Slope to intraplatform basin | This study |
|  | 16dps-19-1 | Wuzhishan Fm. | 358.9-358.4 | 954.81 | 0.02 | Limestone | Slope to intraplatform basin | This study |
|  | 16dps-19-2 | Wuzhishan Fm. | 358.9-358.4 | 915.91 | 0.02 | Limestone | Slope to intraplatform basin | This study |
|  | 16dps-20-1 | Wuzhishan Fm. | 358.9-358.4 | 1269.04 | 0.02 | Limestone | Slope to intraplatform basin | This study |
|  | 16dps-20-2 | Wuzhishan Fm. | 359.3-359.1 | 1040.06 | 0.02 | Limestone | Slope to intraplatform basin | This study |
|  | 16dps-21-1 | Wuzhishan Fm. | 359.3-359.1 | 1130.19 | 0.01 | Limestone | Slope to intraplatform basin | This study |
|  | 16dps-21-2 | Wuzhishan Fm. | 359.3-359.1 | 1039.26 | 0.02 | Limestone | Slope to intraplatform basin | This study |
|  | 16dps-22-1 | Wuzhishan Fm. | 359.3-359.1 | 878.20 | 0.02 | Limestone | Slope to intraplatform basin | This study |
|  | 16dps-22-2 | Wuzhishan Fm. | 359.3-359.1 | 721.01 | 0.02 | Limestone | Slope to intraplatform basin | This study |
|  | 16dps-23-1 | Wuzhishan Fm. | 359.3-359.1 | 879.60 | 0.02 | Limestone | Slope to intraplatform basin | This study |
|  | 16dps-23-2 | Wuzhishan Fm. | 359.3-359.1 | 880.47 | 0.02 | Limestone | Slope to intraplatform basin | This study |
|  | 16dps-24-1 | Wuzhishan Fm. | 359.3-359.1 | 807.05 | 0.02 | Limestone | Slope to intraplatform basin | This study |
|  | 16dps-24-2 | Wuzhishan Fm. | 359.3-359.1 | 1072.21 | 0.02 | Limestone | Slope to intraplatform basin | This study |
|  | 16dps-25-1 | Wuzhishan Fm. | 359.3-359.1 | 662.33 | 0.02 | Limestone | Slope to intraplatform basin | This study |
|  | 16dps-25-2 | Wuzhishan Fm. | 359.3-359.1 | 644.06 | 0.02 | Limestone | Slope to intraplatform basin | This study |

Supplementary Table 3. Sedimentation rates of the Dengying Formation, Rongxian Formation and Wuzhishan Formation.

| Formation | Time | Period | Section | Thickness | Sedimentation Rate | Reference |
| --- | --- | --- | --- | --- | --- | --- |
| Dengying | 551 Ma ~ 541 Ma | 10Ma | Muzhuxia of Hubei Province | 240m | 24 m/Ma | Shen *et al.* (2009)^3^ |
|  |  |  | Liantuo-Wangjiaping of Hubei Province | 645m | 64.5 m/Ma | Zhu *et al.* (2003)^44^ |
|  |  |  | Wuhe of Hubei Province | 234.5m~329.5m | 23.45~32.95 m/Ma | Zhu *et al.* (2003) |
|  |  |  | Wuhe of Hubei Province | ~423m | ~42.3 m/Ma | Chen *et al.* (2013)^7^ |
|  |  |  | Wuhe-Yanjiahe of Hubei Province | ~537m | ~53.7 m/Ma | Duda *et al.* (2014)^45^ |
|  |  |  | Eastern Yangtze Gorges area | 673m | 67.3 m/Ma | Sun (1986)^11^ |
| Rongxian | 372.2 Ma ~ 358.9 Ma | 13.3Ma | Huangluping of Northern Guangxi Province | 1066m | ~80.15 m/Ma | Yin *et al.* (1997)^25^ |
|  |  |  | Longtiao of Guangxi Province | ~414m | ~31.13 m/Ma | Yin *et al.* (1997) |
|  |  |  | Liuzhou of Guangxi Province | ~1866m | ~140.3 m/Ma | Yin *et al.* (1997) |
|  |  | 1.5 Ma | Panlong of Guangxi Province | 9.6m | 6.4 m/Ma | This study |
|  |  | 0.7 Ma | Dazhai of Guizhou Province | 20m | 28.6 m/Ma | This study |
|  |  | 4 Ma | Madao of Guizhou Province | 50m | 12.5 m/Ma | This study |
| Wuzhishan | 359.3 Ma ~ < 357.5 Ma | 1.8 Ma | Duli of Guangxi Province | 7.5m | 4.2 m/Ma | This study |
|  |  | 0.2 Ma | Xiada of Guangxi Province | 0.84 m | 4.2 m/Ma | This study |
|  |  | 1.3 Ma | Daposhang of Guizhou Province | 7.3 m | 5.6 m/Ma | This study |

Supplementary Table 4. TOC, carbonate & siliciclastic components of Dengying Formation, Hubei Province, South China (551 Ma ~ 541 Ma).

| Sample | TOC | Carbonate component | Siliciclastic component |
| --- | --- | --- | --- |
|  | % | % | % |
| SX-01 | 7.78 | 86.01 | 13.99 |
| SX-04 | 4.33 | 86.59 | 13.41 |
| SX-05 | 0.12 | 68.89 | 31.11 |
| SX-07 | 0.64 | 84.85 | 15.15 |
| SX-08 | 0.46 | 79.20 | 20.80 |
| SX-09 | 4.35 | 82.74 | 17.26 |
| SX-10 | 1.05 | 91.35 | 8.65 |
| SX-11 | 0.38 | 70.90 | 29.10 |
| SX-13 | 0.48 | 83.79 | 16.21 |
| SX-15 | 3.55 | 87.08 | 12.92 |
| SX-17 | 2.19 | 81.93 | 18.07 |
| SX-20 | 1.13 | 80.29 | 19.71 |
| SX-23 | 3.36 | 88.68 | 11.32 |
| SX-25 | 1.25 | 90.74 | 9.26 |
| SX-27 | 0.57 | 77.84 | 22.16 |
| SX-28 | 5.31 | 89.22 | 10.78 |
| SX-30 | 0.54 | 87.52 | 12.48 |
| SX-31 | 3.74 | 90.74 | 9.26 |
| SX-32 | 2.90 | 84.56 | 15.44 |
| HNY-01 | 5.91 | 88.22 | 11.78 |
| HNY-04 | 2.12 | 83.83 | 16.17 |
| HNY-06 | 2.78 | 86.63 | 13.37 |
| HNY-09 | 0.66 | 85.62 | 14.38 |
| HNY-13 | 2.83 | 89.12 | 10.88 |
| HNY-17 | 4.50 | 81.62 | 18.38 |
| HNY-22 | 4.13 | 83.00 | 17.00 |
| HNY-24 | 10.17 | 88.96 | 11.04 |
| HNY-28 | 3.98 | 85.96 | 14.04 |
| HNY-30 | 1.15 | 93.89 | 6.11 |
| HNY-35 | 2.09 | 88.30 | 11.70 |
| HNY-38 | 2.35 | 90.08 | 9.92 |
| HNY-40 | 1.08 | 93.53 | 6.47 |
| HNY-41 | 1.65 | 88.28 | 11.72 |
| HNY-51 | 0.57 | 81.58 | 18.42 |
| HNY-53 | 5.10 | 88.32 | 11.68 |
| HNY-54 | 0.55 | 85.88 | 14.12 |
| HNY-56 | 1.92 | 91.62 | 8.38 |
| HNY-59 | 0.62 | 88.81 | 11.19 |
| HNY-62 | 1.12 | 76.24 | 23.76 |
| HNY-67 | 0.70 | 90.74 | 9.26 |
| HNY-80 | 0.35 | 78.21 | 21.79 |
| HNY-85 | 7.40 | 85.66 | 14.34 |
| HNY-92 | 0.96 | 90.04 | 9.96 |
| HNY-93 | 2.13 | 89.92 | 10.08 |
| HNY-94 | 3.56 | 87.98 | 12.02 |
| HNY-98 | 0.19 | 86.01 | 13.99 |
| HNY-100 | 1.59 | 85.15 | 14.85 |

Supplementary Table 5. Carbonate & siliciclastic components of shallow water carbonates (the Rongxian Fm.) in Late Paleozoic (373 Ma ~ 355 Ma), South China.

| Section | Sample | Carbonate component | Siliciclastic component |
| --- | --- | --- | --- |
|  |  | % | % |
| Dazhai Section | **DtZ1-0** | 86.28 | 13.72 |
| (Rongxian Fm.) | **DtZ1-1** | 86.15 | 13.85 |
| (370.7 Ma ~ 370 Ma) | **DtZ1-(-2)** | 80.06 | 19.94 |
|  | **DtZ1-(-3)** | 85.08 | 14.92 |
|  | **DtZ1-c-17** | 87.86 | 12.14 |
|  | **DtZ1-bu-1** | 81.79 | 18.21 |
|  | **DtZ3-4** | 83.51 | 16.49 |
|  | **DtZ3-5** | 86.14 | 13.86 |
|  | **DtZ3-6** | 87.03 | 12.97 |
|  | **DtZ3-7** | 85.39 | 14.61 |
| Panlong Section | **NPL-54** | 97.64 | 2.36 |
| (Rongxian Fm.) | **NPL-51** | 99.07 | 0.93 |
| (373 Ma ~ 372.2 Ma) | **NPL-43** | 97.95 | 2.05 |
|  | **NPL-48** | 99.56 | 0.44 |
|  | **NPL-37** | 98.16 | 1.84 |
|  | **NPL-31** | 98.23 | 1.77 |
|  | **NPL-24** | 97.48 | 2.52 |
|  | **NPL-22** | 100.00 | 0.00 |
|  | **NPL-4-3** | 99.81 | 0.19 |
|  | **NPL-4-23** | 100.00 | 0.00 |
|  | **NPL-4-19** | 97.04 | 2.96 |
|  | **NPL-4-11** | 98.37 | 1.63 |
|  | **NPL-4-5** | 100.00 | 0.00 |
|  | **NPL-4-1** | 99.04 | 0.96 |
| Madao Section | **MD-33** | 100.00 | 0.00 |
| (Rongxian Fm.) | **MD-30** | 99.75 | 0.25 |
| (359 Ma ~ 355 Ma) | **MD-24** | 100.00 | 0.00 |
|  | **MD-16** | 100.00 | 0.00 |
|  | **MD-11** | 100.00 | 0.00 |
|  | **MD-8** | 98.47 | 1.53 |
|  | **MD-1** | 98.21 | 1.79 |

Supplementary Table 6. Carbonate & siliciclastic components of deep water carbonates (the Wuzhishan Formation) in Late Paleozoic (359.3 Ma ~ < 357.5 Ma), South China.

| Section | Sample | Carbonate component | Siliciclastic component |
| --- | --- | --- | --- |
|  |  | % | % |
| Duli Section | DuL10-1a | 85.99 | 14.01 |
| (Wuzhishan Fm.) | DuL10-1b | 86.96 | 13.04 |
| (359.3 Ma ~ 357.5 Ma) | DuL10-2a | 75.64 | 24.36 |
|  | DuL10-2b | 71.49 | 28.51 |
|  | DuL10-2c | 91.04 | 8.96 |
|  | DuL-14a | 71.15 | 28.85 |
|  | DuL-14b | 67.64 | 32.36 |
|  | DuL-15a | 88.58 | 11.42 |
|  | DuL-15b | 88.34 | 11.66 |
|  | DuL-16a | 77.58 | 22.42 |
|  | DuL-16b | 76.03 | 23.97 |
|  | DuL-16c | 83.20 | 16.80 |
|  | DuL-16d | 83.16 | 16.84 |
|  | DuL-17a | 84.28 | 15.72 |
|  | DuL-17b | 83.81 | 16.19 |
|  | DuL-17c | 84.63 | 15.37 |
|  | DuL-18a | 82.34 | 17.66 |
|  | DuL-18b | 86.75 | 13.25 |
|  | DuL-18c | 78.94 | 21.06 |
|  | DuL-19a | 87.97 | 12.03 |
|  | DuL-19b | 87.49 | 12.51 |
|  | DuL-20a | 83.56 | 16.44 |
|  | DuL-20b | 86.78 | 13.22 |
| Daposhang Section | 16dps-10-1 | 92.23 | 7.77 |
| (Wuzhishan Fm.) | 16dps-10-2 | 80.58 | 19.42 |
| (359.3 Ma ~ 358 Ma) | 16dps-11-1 | 91.77 | 8.23 |
|  | 16dps-11-2 | 80.12 | 19.88 |
|  | 16dps-12-1 | 92.50 | 7.50 |
|  | 16dps-12-2 | 86.63 | 13.37 |
|  | 16dps-13-1 | 97.68 | 2.32 |
|  | 16dps-13-2 | 93.46 | 6.54 |
|  | 16dps-14-1 | 84.32 | 15.68 |
|  | 16dps-14-2 | 89.02 | 10.98 |
|  | 16dps-15-1 | 96.62 | 3.38 |
|  | 16dps-15-2 | 86.86 | 13.14 |
|  | 16dps-16-1 | 87.71 | 12.29 |
|  | 16dps-16-2 | 91.89 | 8.11 |
|  | 16dps-17-1 | 87.07 | 12.93 |
|  | 16dps-17-2 | 91.05 | 8.95 |
|  | 16dps-18-1 | 85.69 | 14.31 |
|  | 16dps-18-2 | 87.56 | 12.44 |
|  | 16dps-19-1 | 87.68 | 12.32 |
|  | 16dps-19-2 | 86.64 | 13.36 |
|  | 16dps-20-1 | 87.05 | 12.95 |
|  | 16dps-20-2 | 75.64 | 24.36 |
|  | 16dps-21-1 | 94.31 | 5.69 |
|  | 16dps-21-2 | 96.91 | 3.09 |
|  | 16dps-22-1 | 93.07 | 6.93 |
|  | 16dps-22-2 | 83.22 | 16.78 |
|  | 16dps-23-1 | 93.66 | 6.34 |
|  | 16dps-23-2 | 95.63 | 4.37 |
|  | 16dps-24-1 | 97.70 | 2.30 |
|  | 16dps-24-2 | 97.24 | 2.76 |
|  | 16dps-25-1 | 98.90 | 1.10 |
|  | 16dps-25-2 | 95.41 | 4.59 |
| Xiada Section | xd12-20-1-1 | 99.95 | 0.05 |
| (Wuzhishan Fm.) | xd12-20-1-2 | 100.00 | 0.00 |
| (359.3 Ma ~ 359.1 Ma) | xd12-20-2-1 | 96.52 | 3.48 |
|  | xd12-20-2-2 | 99.18 | 0.82 |
|  | xd12-21-1-1 | 100.00 | 0.00 |
|  | xd12-21-1-2 | 100.00 | 0.00 |
|  | xd12-21-2-1 | 100.00 | 0.00 |
|  | xd12-21-2-2 | 100.00 | 0.00 |
|  | xd12-22-1-1 | 100.00 | 0.00 |
|  | xd12-22-1-2 | 97.17 | 2.83 |
|  | xd12-22-2-1 | 100.00 | 0.00 |
|  | xd12-22-2-2 | 100.00 | 0.00 |
|  | xd12-23-1-1 | 100.00 | 0.00 |
|  | xd12-23-1-2 | 100.00 | 0.00 |
|  | xd12-23-2-1 | 100.00 | 0.00 |
|  | xd12-23-2-2 | 100.00 | 0.00 |
|  | xd12-24-1-1 | 100.00 | 0.00 |
|  | xd12-24-1-2 | 100.00 | 0.00 |
|  | xd12-24-2-1 | 100.00 | 0.00 |
|  | xd12-24-2-2 | 100.00 | 0.00 |
|  | xd12-25-1-1 | 99.51 | 0.49 |
|  | xd12-25-1-2 | 100.00 | 0.00 |
|  | xd12-25-2-1 | 100.00 | 0.00 |
|  | xd12-25-2-2 | 100.00 | 0.00 |
|  | xd12-26L-1 | 100.00 | 0.00 |
|  | xd12-26L-2 | 99.36 | 0.64 |
|  | xd12-26U-1 | 98.08 | 1.92 |
|  | xd12-26U-2 | 99.78 | 0.22 |

**Supplementary References**

1 Condon, D. *et al.* U-Pb ages from the neoproterozoic Doushantuo Formation, China. *Science* **308**, 95-98, (2005).

2 Zhao, Z. *et al.* *The Sinian System of Hubei*. Vol. 205 (China University of Geosciences Press, Wuhan, 1988).

3 Shen, B., Xiao, S. H., Zhou, C. M. & Yuan, X. L. Yangtziramulus Zhangi New Genus and Species, a Carbonate-Hosted Macrofossil from the Ediacaran Dengying Formation in the Yangtze Gorges Area, South China. *J. Paleontol.* **83**, 575-587, (2009).

4 Meyer, M. *et al.* Interactions between Ediacaran animals and microbial mats: Insights from Lamonte trevallis , a new trace fossil from the Dengying Formation of South China. *Palaeogeogr. Palaeoclimatol. Palaeoecol.* **396**, 62-74, (2014).

5 Zhu, M. Y., Zhang, J. M. & Yang, A. H. Integrated Ediacaran (Sinian) chronostratigraphy of South China. *Palaeogeography Palaeoclimatology Palaeoecology* **254**, 7-61, (2007).

6 Zhu, M. Y. *et al.* Carbon isotope chemostratigraphy and sedimentary facies evolution of the Ediacaran Doushantuo Formation in western Hubei, South China. *Precambrian Res.* **225**, 7-28, (2013).

7 Chen, Z. *et al.* Trace fossil evidence for Ediacaran bilaterian animals with complex behaviors. *Precambrian Res.* **224**, 690-701, (2013).

8 Chen, P. Discovery of Lower Cambrian small shelly fossils from Jijiapo, Yichang, west Hubei and its significance. *Prof. Pap. Stratigra. Palaeontol.* **13**, 49-66, (1984).

9 Yao, J. X., Xiao, S. H., Yin, L. M., Li, G. X. & Yuan, X. L. Basal Cambrian microfossils from the Yurtus and Xishanblaq formations (Tarim, north-west China): Systematic revision and biostratigraphic correlation of Micrhystridium-like acritarchs. *Palaeontology* **48**, 687-708, (2005).

10 Dong, L. *et al.* Basal Cambrian Microfossils from the Yangtze Gorges Area (South China) and the Aksu Area (Tarim Block, Northwestern China). *J. Paleontol.* **83**, 30-44, (2009).

11 Sun, W. Late precambrian pennatulids (sea pens) from the eastern Yangtze Gorge, China: Paracharnia gen. nov. *Precambrian Res.* **31**, 361–375, (1986).

12 Weber, B., Steiner, M. & Zhu, M. Precambrian–Cambrian trace fossils from the Yangtze Platform (South China) and the early evolution of bilaterian lifestyles. *Palaeogeogr. Palaeoclimatol. Palaeoecol.* **254**, 328-349, (2007).

13 Gnilovskaya, M. B. The oldest aquatic plants of the Vendian of the Russian Platform (late Precambrian). *Paleontol. J.* **5**, 372-378, (1971).

14 Ding, Q. & Chen, Y. Discovery of soft metazoan from the Sinian System along eastern Yangtze Gorge, Hubei. *J. Wuhan College Geol.* **2**, 53-57, (1981).

15 Xiao, S., Shen, B., Zhou, C., Xie, G. & Yuan, X. A uniquely preserved Ediacaran fossil with direct evidence for a quilted bodyplan. *Proc. Natl Acad. Sci. USA* **102**, 10227-10232, (2005).

16 Chen, Z. *et al.* New Ediacara fossils preserved in marine limestone and their ecological implications. *Sci. Rep.* **4**, 4180, (2014).

17 Steiner, M., Mehl, D., Reitner, J. & Erdtamnn, B. D. Oldest entirely preserved sponges and other fossils from the Lowermost Cambrian and a new facies reconstruction of the Yangtze platform (China). *Openaccess* **9**, 293-329, (1993).

18 Steiner, M. & Reitner, J. Evidence of organic structures in Ediacara-type fossils and associated microbial mats. *Geology* **29**, 1119-1122, (2001).

19 Gehling, J. G. Microbial mats in terminal Proterozoic siliciclastics: Ediacaran death masks. *Palaios* **14**, 40-57, (1999).

20 Chen, Z., Chen, X., Zhou, C., Yuan, X. & Xiao, S. Late Ediacaran trackways produced by bilaterian animals with paired appendages. *Sci Adv* **4**, (2018).

21 Chen, D., Tucker, M. E., Jiang, M. & Zhu, J. Long‐distance correlation between tectonic‐controlled, isolated carbonate platforms by cyclostratigraphy and sequence stratigraphy in the Devonian of South China. *Sedimentology* **48**, 57-78, (2001).

22 Chen, D., Tucker, M. E., Zhu, J. & Jiang, M. Carbonate sedimentation in a starved pull-apart basin, Middle to Late Devonian, southern Guilin, South China. *Basin Res* **13**, 141-167, (2001).

23 Chen, D. Z., Tucker, M. E., Zhu, J. Q. & Jiang, M. S. Carbonate platform evolution: from a bioconstructed platform margin to a sand-shoal system (Devonian, Guilin, South China). *Sedimentology* **49**, 737-764, (2002).

24 Nie, T. *et al.* Age and distribution of the Late Devonian brachiopod genus Dzieduszyckia Siemiradzki, 1909 in southern China. *Palaeoworld* **25**, 600-615, (2016).

25 Yin, B. *et al.* *Lithostratigraphy in Guangxi*. (China Univ. Geosci. Press, 1997).

26 Severmann, S., McManus, J., Berelson, W. M. & Hammond, D. E. The continental shelf benthic iron flux and its isotope composition. *Geochim. Cosmochim. Acta* **74**, 3984-4004, (2010).

27 McManus, J., Berelson, W. M., Coale, K. H., Johnson, K. S. & Kilgore, T. E. Phosphorus regeneration in continental margin sediments. *Geochim. Cosmochim. Acta* **61**, 2891-2907, (1997).

28 Friedrich, J. *et al.* Benthic Nutrient Cycling and Diagenetic Pathways in the North-western Black Sea. *Estuar. Coast. Shelf Sci.* **54**, 369-383, (2002).

29 Berelson, W. *et al.* A time series of benthic flux measurements from Monterey Bay, CA. *Cont. Shelf Res.* **23**, 457-481, (2003).

30 Pakhomova, S. V. *et al.* Fluxes of iron and manganese across the sediment–water interface under various redox conditions. *Mar. Chem.* **107**, 319-331, (2007).

31 Homoky, W. B. *et al.* Dissolved oxygen and suspended particles regulate the benthic flux of iron from continental margins. *Mar. Chem.* **134-135**, 59-70, (2012).

32 Marsay, C. M. *et al.* Estimating the benthic efflux of dissolved iron on the Ross Sea continental shelf. *Geophys. Res. Lett.* **41**, 7576-7583, (2014).

33 Noffke, A. *et al.* Benthic iron and phosphorus fluxes across the Peruvian oxygen minimum zone. *Limnol. Oceanogr.* **57**, 851-867, (2012).

34 Elrod, V. A., Berelson, W. M., Coale, K. H. & Johnson, K. S. The flux of iron from continental shelf sediments: A missing source for global budgets. *Geophys. Res. Lett.* **31**, (2004).

35 Dale, A. W. *et al.* A revised global estimate of dissolved iron fluxes from marine sediments. *Glob Biogeochem Cycles* **29**, 691-707, (2015).

36 Di Lorenzo, F., Burgos-Cara, A., Ruiz-Agudo, E., Putnis, C. V. & Prieto, M. Effect of ferrous iron on the nucleation and growth of CaCO3 in slightly basic aqueous solutions. *CrystEngComm* **19**, 447-460, (2017).

37 Dromgoole, E. L. & Walter, L. M. Iron and manganese incorporation into calcite: Effects of growth kinetics, temperature and solution chemistry ☆. *Chem Geol* **81**, 311-336, (1990).

38 Sperling, E. A. *et al.* Statistical analysis of iron geochemical data suggests limited late Proterozoic oxygenation. *Nature* **523**, 451-454, (2015).

39 Kump, L. R. The rise of atmospheric oxygen. *Nature* **451**, 277, (2008).

40 Kaiho, K. Benthic Foraminiferal Dissolved-Oxygen Index and Dissolved-Oxygen Levels in the Modern Ocean. *Geology* **22**, 719-722, (1994).

41 Sperling, E. A. *et al.* Oxygen, ecology, and the Cambrian radiation of animals. *Proc Natl Acad Sci USA* **110**, 13446-13451, (2013).

42 Chen, D. & Tucker, M. E. The Frasnian–Famennian mass extinction: insights from high-resolution sequence stratigraphy and cyclostratigraphy in South China. *Palaeogeogr., Palaeoclimatol., Palaeoecol.* **193**, 87-111, (2003).

43 Poulton, S. W. & Raiswell, R. The low-temperature geochemical cycle of iron: From continental fluxes to marine sediment deposition. *Am. J. Sci.* **302**, 774-805, (2002).

44 Zhu, M. *et al.* Sinian-Cambrian stratigraphic framework for shallow-to deep-water environments of the Yangtze Platform: an integrated approach. *Prog. Nat. Sci.* **13**, 951-960, (2003).

45 Duda, J. P. *et al.* Geobiology of a palaeoecosystem with Ediacara-type fossils: The Shibantan Member (Dengying Formation, South China). *Precambrian Res.* **255**, 48-62, (2014).
